# SUMOylation of SARS-CoV-2 spike protein is a key target for broad-spectrum antiviral therapy

**DOI:** 10.7150/thno.111256

**Published:** 2025-05-25

**Authors:** Xinyu Wang, Gaowei Hu, Yupeng Shao, Zhongwei Dong, Lina Liu, Yixuan Wang, Yaxi Xie, Nuoya Yu, Caixia Zhu, Fang Wei, Yuping Jia, Yuyan Wang, Qiliang Cai

**Affiliations:** 1 MOE/NHC/CAMS Key Laboratory of Medical Molecular Virology, Shanghai Institute of Infections Disease and Biosecurity, Shanghai Frontiers Science Center of Pathogenic Microorganisms and Infection, School of Basic Medical Sciences, Qidong-Fudan Innovative Institute of Medical Science, Shanghai Medical College, Fudan University, Shanghai 200032, P. R. China; 2 ShengYushou Center of Cell Biology and Immunology, Joint International Research Laboratory of Metabolic & Development Sciences, School of Life Sciences and Biotechnology, Shanghai Jiao Tong University, Shanghai 200240, P. R. China; 3 Expert Workstation, Baoji Central Hospital, Baoji 721008, P. R. China.; 4 Shandong Academy of Pharmaceutical Sciences, Jinan, 250100, P. R. China

**Keywords:** SUMOylation, SARS-CoV-2, Spike protein, Antiviral peptides

## Abstract

**Background:** Dynamic SUMO modifications play crucial roles in orchestrating cellular response to various stimuli, including viral infection, and hold significant therapeutic potential. The Spike (S) protein, a surface glycoprotein of SARS-CoV-2 (a global health threat), serves as the key mediator for viral entry and is a critical target for drug development. However, the function of SUMOylation in the Spike protein remains largely unclear.

**Methods:** The SUMO modification of Spike was assessed by immunoprecipitation (IP), denatured IP and immunoblotting assays in lung epithelial cells or SUMO deficient cell line models. The effect of Spike SUMOylation on viral infection was explored by site-direct mutation, cell-to-cell transmission, cell-free infection, quantitative PCR and immunofluorescence staining experiments. The role of Spike SUMOylation-derived peptides was investigated using viral intranasally infection, immunohistochemistry assay in transgenic mouse model.

**Results:** SARS-CoV-2 infection triggers the relocation of SUMO1 to the cytoplasm and SUMO2 to the perinuclear region. Notably, SUMO1 knockout increases Spike trimer formation and its co-localization with SUMO2 at perinuclear puncta, which facilitates virion particle release. SUMO2 knockout leads to enhanced Spike cleavage and promotes viral cell-to-cell transmission. Further bioinformatic and immuno-precipitation analyses reveal that the Spike protein contains highly conserve SUMO-interacting motifs (SIMs) and selectively promotes either SUMO1 (via SIM1) or SUMO2 (via SIM3/4) modifications on lysine residues 129 and 1269, respectively. Importantly, these modifications can be efficiently disrupted by the SIM2 motif. A cell-penetrating peptide (cpSIM2), derived from the SIM2 motif, exhibits robust and broad-spectrum inhibitory activity against SARS-CoV-2 variants infection *in vitro* and in hACE2-transgenic mice model.

**Conclusions:** This study uncovers critical features of SUMOylation in regulating Spike-mediated viral spread and pathogenesis, providing a potential broad-spectrum therapeutic target for drug development against emerging SARS-CoV-2 infection.

## Introduction

Small ubiquitin-like modifier (SUMO) modification, or SUMOylation, serves as a rapid and adaptive mechanism for cells to regulate proteome functionality in response to various stimuli, including promyelocytic leukemia protein-nucleosome (PML-NB) formation, DNA damage response and pathogen invasion 1. SUMO proteins exhibit remarkable evolutionary conservation across all eukaryotes 2. In humans, five SUMO paralogs (SUMO1-5) have been identified through genomic studies 3. Among these, SUMO1, SUMO2, and SUMO3 are ubiquitously expressed and extensively characterized in various human tissues 3, representing the primary focus of this investigation. In contrast, SUMO4 demonstrates tissue-restricted expression patterns, predominantly localized to lymphoid organs (e.g., lymph nodes and spleen) and kidneys, with aberrant expression linked to diabetes pathogenesis 4, 5, while SUMO5 displays even narrower tissue specificity, primarily observed in peripheral blood leukocytes and testicular tissue 6. Due to their 98% homology, SUMO2 and SUMO3 are often collectively referred to as SUMO2/3 7. SUMO1 and SUMO2/3 modifications exhibit distinct physiological functions, with SUMO2/3 preferentially modifying host and viral proteins under stress conditions 8. It has been documented that a target protein can undergo different types of SUMO modifications at various lysine (K) residues and can bind to other proteins through SUMO-interacting motifs (SIMs), providing an additional interaction platform for the recruitment of downstream effector proteins 9. This dynamic interplay regulates the nature of SUMO modification at specific sites. Increasing evidence shows that the SUMO system is pivotal in the host response to viral infection 10. Many viral proteins not only serve as substrates for this reversible PTM, but also modulate the host SUMO pathway 11. Thus, specific inhibitors targeting viral protein SUMOylation offer a promising strategy for therapeutic development. Recent research has highlighted the impact of SUMO modification on coronavirus infection, demonstrating that both SARS-CoV and SARS-CoV-2 nucleocapsid (N) proteins can undergo SUMO modification to promote oligomerization 12, 13. Additionally, SUMO3 modification of the ACE2 receptor facilitates SARS-CoV-2 infection 14. Despite these findings, the SUMOylation of other SARS-CoV-2 viral proteins remain largely unexplored, and therapeutic agents targeting viral protein SUMOylation have not yet to be developed so far.

The SARS-CoV-2 Spike (S) glycoprotein, a trimer structure on the viral envelope surface, is pivotal for viral entry into target cells 15. The Spike protein comprises two subunits: S1 and S2. The S1 subunit contains the receptor-binding domain (RBD), responsible for binding to the ACE2 receptor, while the S2 subunit facilitates the fusion of viral and cellular membranes 16, 17. The S1/S2 junction includes a multibasic cleavage site recognized by the cellular protease furin. Cleavage at this site is essential for Spike-mediated cell-to-cell transmission and viral invasion 18. Within infected cells, the Spike protein undergoes furin cleavage during trafficking through the Golgi and anti-Golgi network, forming non-covalently linked S1 and S2 subunits 19. This cleavage exposes the S1 submit for more efficient interaction with ACE2 and facilitates the exposure and subsequent cleavage of S2, leading to conformational rearrangement 19. In addition to assembly into virus particles, the S protein can mediate viral cell-to-cell transmission when expressed solely on the cell surface 19. Thus, intracellular trafficking and cleavage of the S protein dictate the modes of viral infection. Similar to other viral surface proteins, the S protein undergoes diverse PTMs, such as glycosylation, which influence its cleavage and incorporation into viral particles 20-23. However, the SUMOylation of viral surface proteins and its functional implications have not been previously reported. Given the rapid mutation and evolution of SARS-CoV-2, the S protein, which serves as a target for numerous COVID-19 treatment strategies including antibodies, vaccines, and targeted drugs, is prone to rendering these therapies ineffective 24, 25. Consequently, the development of broad-spectrum targeted therapeutic agents against the S protein is of utmost necessity.

In this study, we found that SARS-CoV-2 infection induces the relocation of SUMO1 to the cytoplasm, and SUMO2 to the perinuclear region. Particularly, the Spike protein undergoes SUMOylation and contains specific SUMO-interacting motifs (SIM1 and SIM3/4) that selectively facilitate SUMO1 and SUMO2 modifications on lysine residues 129 and 1269, respectively. These modifications lead to either viral cell-to-cell transmission or virion particle release. Importantly, we have demonstrated that a cell-penetrating peptide (cpSIM2) derived from the SIM2 motif exhibits potent and broad-spectrum inhibitory activity against SARS-CoV-2 infection both *in vitro* and *in vivo*, highlighting its potential as a novel therapeutic strategy for combating emerging coronavirus strains and its significance in the development of broad-spectrum antiviral therapies.

## Materials and Methods

**Cell Culture and Transfection —** Human Embryonic Kidney 293T (HEK293T), A549-hACE2 (a gift from Dr. Rong Zhang at the Fudan University), and Calu3 cells were maintained in Dulbecco's modified Eagle's medium (DMEM) supplemented with 10% fetal bovine serum (FBS), and 100 U/mL of penicillin-streptomycin-gentamycin. All cell lines were incubated at 37 °C in a humidified environmental incubator with 5% CO_2_. For transfections, HEK 293T cells were grown to 60-70% confluence before being transfected with plasmid DNA and polyethyleneimine (PEI; MW40000, Yeasen catalog no.40816ES03) at a ratio of 1 μg DNA to 3 μL PEI (1mg/mL). A549-hACE2 cells were transfected using Lipofectamine 3000 (Thermo Fisher Scientific) according to the manufacturer's protocol. Plasmid DNA was introduced first, followed by poly(I:C) (Invivogen) transfection 24 h later.

**Construction of SUMO-Knockout Cells —** To generate SUMO-knockout (SUMO-KO) cells, oligonucleotides encoding single-guide RNAs (sgRNAs) targeting SUMO1 (5'-GTGTTCCAATGAATTCACTC-3'), SUMO2 (5'-GAAAGC CTATTGTGAACGAC-3'), and SUMO3 (5'- GGCCGGGCAGGACGGCTCCG-3') were inserted into the plentiCRISPR-V2 vector, respectively. The resulting plentiCRISPR-V2 vectors, each carrying a specific sgRNA, were co-transfected with lentivirus packaging plasmids into HEK293T cells for 48 h to produce lentiviruses. These viruses were then used to infect A549-hACE2 or HEK293T cells, followed by selection with 1 μg/mL puromycin. Single-cell clones were isolated by limiting dilution in 96-well plates and expanded. Clones were screened for SUMO1, SUMO2, and SUMO3 expression by immunoblotting, and knockout efficiency was confirmed by PCR.

**Virus Infection and Drug Delivery —** The SARS-CoV-2 wild-type SH01 strain (GenBank accession no. MT121215), Delta variant, and Omicron BA.1 and BA.2 subvariants were obtained from the Chinese Center for Disease Control and Prevention, isolated from patients in Shanghai, China. For infection experiments, SARS-CoV-2 was diluted in DMEM containing 2% FBS to achieve a multiplicity of infection (MOI) of 1 or 0.1 and used to infect A549-hACE2 and Calu3 cells. After infection, cells were incubated at 37 °C with 5% CO_2_ for 24 h to allow virus replication. For immediate drug treatment, cells were incubated with virus for 1 h, unbound virus was removed by washing, and cells were treated with respective peptide drugs. All experiments involving SARS-CoV-2 were conducted in a BSL-3 facility at Fudan University.

**Plasmids —** The plasmids S-pA3M (S) expressing full length myc-tagged Spike protein was constructed by ligating the Spike fragment from S-pCAGGS-nCoV 26 (kindly provided from Dr. Rong Zhang at Fudan University) into the pA3M (or pcDNA3.1) vector digested with *Bam*HI and *Eco*RI. SUMOylation mutants (K129R, K462E, K933R, K1149R, and K1269R) and SIM mutants (mSIM1, mSIM1', mSIM2, mSIM3, and mSIM4) of Spike were constructed by PCR site-directed mutagenesis using S-pA3M as a template. The plasmid ORF9b-pA3F was created by PCR amplification from SARS-CoV-2 genome cDNA, followed by digestion with *Bam*HI and *Eco*RI, and ligation into the pA3F vector. Primers for constructing these plasmids are listed in Supplementary Table S1. HA-SUMO1, HA-SUMO2, and HA-SUMO3 plasmids were stored in the laboratory 27. The plasmids expressing the Spike protein encoded by SARS-CoV-1 (kindly provided from Dr. Dongming Zhou at Tianjin Medical University) or SARS-CoV-2 Wuhan-Hu-1, XBB.1.5, and JN.1 (Kindly provided by Dr. Lu Lu from Fudan University) were used.

**Immunoprecipitation, Denatured Immunoprecipitation, and Immuno- blotting —** Immunoprecipitation (IP), denatured IP, and immuno-blotting (IB) assays were performed as previously described 28. Membrane-bound proteins of interest were detected using an Odyssey Infrared scanner and analyzed with Li-Cor Biosciences software. Antibodies used included: SUMO1 (10329-1-AP, Proteintech), SUMO2/3 (11251-1-AP, Proteintech), SARS-CoV-2 Spike S1 (A20834, Abclonal), SARS-CoV-2 Spike S2 (A20284, Abclonal), SARS-CoV-2 S_600-700_ (A20137, Abclonal), SARS-CoV-2 N (a gift from Dr. Rong Zhang at Fudan University) 26, HA (51064-2-AP, Proteintech), and α-Tubulin (66031-1-Ig, Proteintech). All antibodies were used according to the manufacturers' instructions.

**Immunofluorescence —** Infected or transfected cells were washed twice with PBS, fixed with 4% paraformaldehyde (PFA) in PBS for 20 min, and permeabilized with 0.1% Triton X-100 for 30 min. Cells were then incubated overnight at 4 °C with primary antibodies: SARS-CoV-2 Nucleoprotein (A18797, Abclonal, 1:500), SARS-CoV-2 Spike S1 (A20834, Abclonal, 1:200), SUMO1 (Y299, Abcam, 1:250), SUMO2/3 (ab81371, Abcam, 1:250), PML (SC-966, Santa Cruz, 1:15), STUB1 (EPR4447, Abcam, 1:200), LaminB1(66095-1-Ig, Proteintech, 1:200), RanGAP1 (A13347, Abclonal,1:200), RanBP2 (SC-74518, Santa Cruz, 1:15), FLAG (M2, Sigma, 1:1000), and FLAG (80010-1-RR, Proteintech,1:800). After three washes, cells were incubated with secondary antibodies for 1 h at room temperature. DNA was counterstained with DAPI (4′,6′-diamidino-2-phenylindole), and coverslips were mounted with p-phenylenediamine. Images were captured using a Leica SP8 confocal microscope and an Operetta High Content Imaging System (PerkinElmer). Subsequent image processing was conducted using ImageJ v2.0.0 (http://rsb.info.nih.gov/ij/) and the PerkinElmer Harmony high-content analysis software v4.9.

**Quantitative PCR —** Total RNA was extracted from cells, supernatants, or tissues using TRIzol reagent (Thermo Fisher). Virus RNA was reverse-transcribed and quantified using the HiScript II One Step RT-qPCR SYBR Green Kit (Q221-01, Vazyme) on a CFX Connect Real-Time System (Bio-Rad). Relative mRNA levels of target genes were calculated using the 2^-△△Ct^ method, with GAPDH as an internal control. Viral titers were expressed as log_10_ viral copies per gram, based on a standard curve. The concentration of the standard DNA was measured in picograms (pg) per microliter (µL). Primer sequences for the indicated gene products are provided in Supplementary Table S2.

**Cell-to-Cell Transmission and Cell-Free Infection Assays —** Following the protocol as described previously by Zeng et al. 29, we utilized a lentiviral vector system to assess cell-to-cell transmission and cell-free Infection. The system employs an anti-sense reporter gene, Nluc, which is expressed only in infected target cells, not in virus-producing cells. SARS-CoV-2 Spike protein, NL4.3-inGluc (kindly provided from Dr. Cong Zeng at Fudan University), and eGFP were co-transfected into donor cells (293T WT, SUMO1-KO, SUMO2-KO, SUMO3-KO). At 24 h post-transfection, donor cells were trypsinized, washed to remove EDTA, and co-cultured with target cells (A549-hACE2) at a 1:1 ratio in 24-well plates for 72 h. Nluc activity in the supernatants was measured at 24, 48, and 72 h. For cell-free Infection, an equivalent number of transfected donor cells were seeded into new 24-well plates and incubated for the same duration as the cell-to-cell transmission assays (typically 72 h). Supernatants were collected and used to infect target cells, ensuring comparable cell number and densities. Nluc activity was measured at the same time points, and cell-to-cell transmission values were calculated by subtracting cell-free infection values from co-cultured cell Nluc activity. Nluc activity was assayed using the Nano-Glo® Luciferase Reporter Gene Assay System (N1110, Promega).

**Interfering Peptides —** Peptides were synthesized by Genscript Biotech at >95% purity and stored at -20 °C in powder aliquots of 1 mg. Peptides were dissolved in PBS when used. For *in vivo* experiments, peptides were administered via intraperitoneal injection (i.p.) into mice.

**Animal Experiments —** Age-matched 6- to 8-week-old female K18-hACE2-2A-CreERT2 C57BL/6J mice were obtained from Cyagen Biosciences Inc. (Jiangsu, China). Mice were intranasally inoculated with 1x10^4^ plaque-forming units (PFU) of SARS-CoV-2 Omicron BA.5.2. Following inoculation, the mice were treated with either 25 mg/kg of cpCtrl, or cpSIM, all diluted in PBS, for five consecutive days. On the fifth day post-infection, the mice were euthanized, and their lungs were harvested for analysis of viral mRNA abundance, immunohistochemistry, and hematoxylin and eosin staining. All the animal experiments involving SARS-CoV-2 were conducted in the BSL-3 laboratory at Fudan University (Shanghai, China). The experiment protocol (No. 20210916-001) was approved by the Animal Ethics Committee of School of Basic Medical Sciences at Fudan University. CpSIM2 toxicity was evaluated using 6- to 8-week-old male C57BL/6J mice. Mice were intraperitoneally injected with PBS or cpSIM2 at doses of 12.5, 25, or 50 mg/kg daily for 4 days. On day 5 post-treatment, mice were euthanized, and blood and organs were collected for biochemical examination, complete blood count and histopathological analysis.

**Histology and Immunohistochemistry Analysis —** Lung tissues from the mice were dissected, fixed in 4% formalin, embedded in paraffin, sectioned into 5 μm slices, and stained with hematoxylin and eosin. For immunohistochemistry, lung tissue sections from each group were stained using an anti-SARS-CoV-2 N protein antibody (A18797, Abclonal, 1:200). All histological and immunohistochemical analyses were performed by Servicebio Company. Tissue sections were imaged using a WS-10 Zhiyue panoramic scanner, and the percentage of positive cells was quantified using ImageJ software.

**Statistical analysis —** Statistical significance was determined using one-way ANOVA or two-way ANOVA with GraphPad Prism software. Each experiment was repeated at least three times unless otherwise specified, and data are presented as the mean ± standard deviation (SD).

## Results

### SARS-CoV-2 Infection Hijacks Host SUMO System

**Figure 1:**
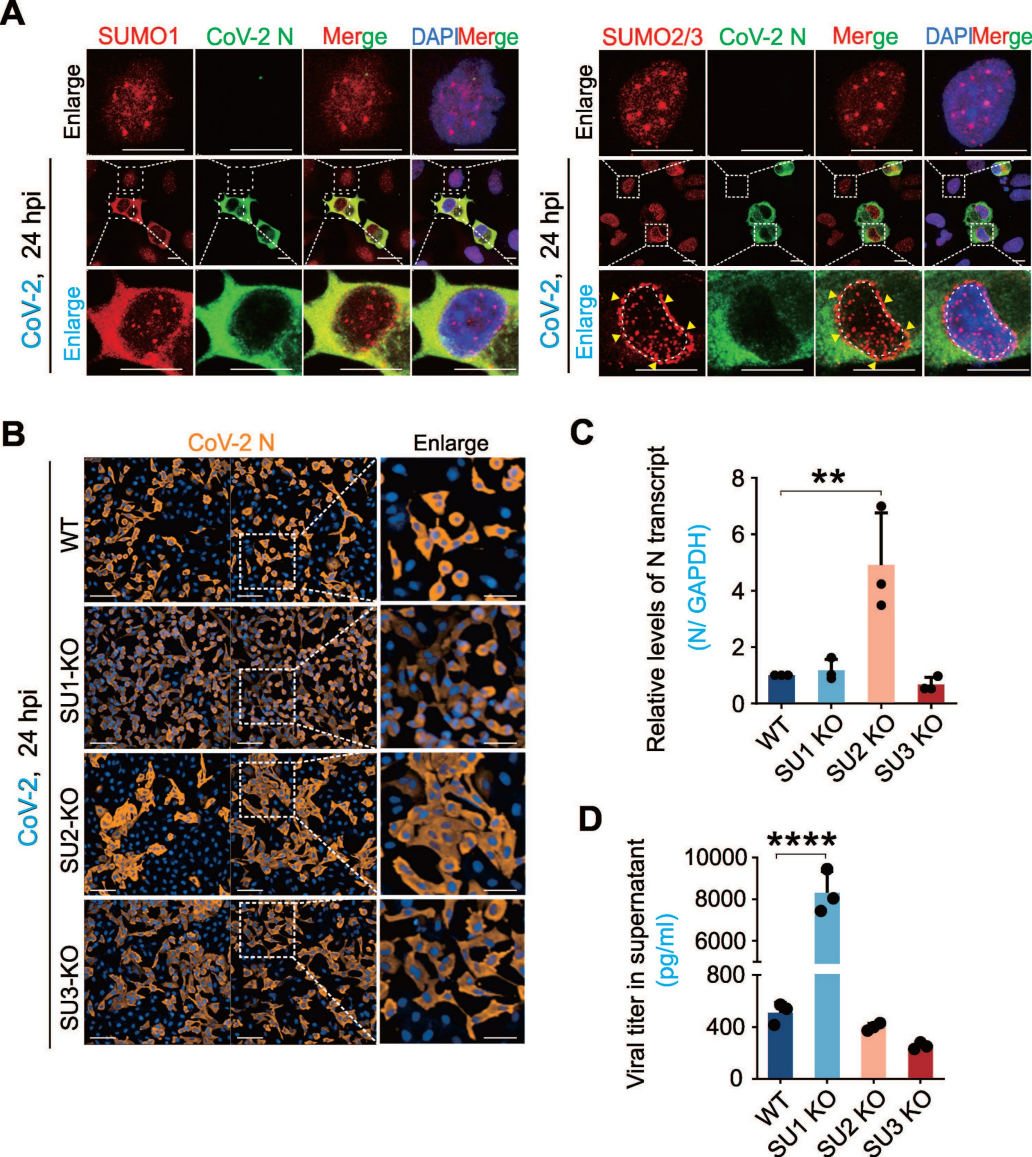
**The interplay between SARS-CoV-2 infection and host SUMOylation. (A) Subcellular localization of host SUMO proteins in response to CoV-2 infection.** A549-hACE2 cells were infected with CoV-2 (strain SH01) at a multiplicity of infection (MOI) of 1 for 24 h, followed by immunofluorescence analysis using the antibodies as indicated in figure. The scale bar represents 5 μm. The enlarged views of infected and non-infected cells were shown. Arrows indicate the perinuclear localization of SUMO2/3. Nuclei were stained with DAPI. (**B**) Impact of SUMO subtype knockouts on CoV-2 infection. A549-hACE2 cells, either wild-type (WT) or with knockouts of SUMO1(SU1-KO), SUMO2 (SU2-KO), or SUMO3 (SU3-KO), were infected with CoV-2 (MOI = 1) for 24 h, followed by immunofluorescence using antibodies against the CoV-2 N protein. Enlarged views of cell morphology after infection are shown in the right panels. Scale bar, 20 μm. (**C**) Influence of SUMO subtype knockouts on CoV-2 replication in infected cells. Total RNA extracts from the A549-hACE2 cells infected with CoV-2 in panel **B**, were analyzed by reverse transcription-quantitative PCR (qPCR) targeting the CoV-2 N gene to assess viral replication. (**D**) Influence of SUMO subtype knockouts on virion particles production. Supernatants from the same A549-hACE2 cells infected with CoV-2 in panel **B**, were also analyzed by RT-qPCR targeting the CoV-2 N gene to evaluate virion particle production. Data represents three independent replicates (n = 3). Each bar indicates the mean ± standard deviation. **p < 0.01; ****p < 0.0001.

To investigate whether the host SUMO system is impaired during SARS-CoV-2 infection, we examined the intracellular distribution of SUMO1 and SUMO2/3 in A549-hACE2 cells (a human non-small cell lung cancer cell line engineered to express human ACE2) with infection of the original strain of SARS-CoV-2 (SH01). Interestingly, we found that different from SUMO1 and SUMO2/3 typically localizes as discrete intra-nuclear punctate foci 1, SARS-CoV-2 infection triggered a significant redistribution: SUMO1 accumulated in the cytoplasm, and SUMO2/3 exhibited a perinuclear punctate pattern (Figure 1A). To our knowledge, this perinuclear punctate distribution of SUMO2/3 is a novel phenomenon not previously observed in other pathogen infections. Additionally, SUMO-related proteins such as PML and STUB1 also showed cytoplasmic redistribution after SARS-CoV-2 infection, displaying distinct localization patterns compared to uninfected cells (Supplementary Figure S1A).

To explore the role of SUMOylation on SARS-CoV-2 infection, we used CRISPR-Cas9 to generate A549-hACE2 cell lines with targeted knockouts (KO) of SUMO1, SUMO2, and SUMO3. Immunoblotting confirmed the knockout efficiency, and sequencing validated the bio-modal knockout profile of SUMO2-KO cells, indicating haploid knockout status (Supplementary Figure S1B-C), due to the high homology between SUMO2 and SUMO3 that could not be differentiation by antibodies. Infection of these cell lines with SARS-CoV-2 (SH01) revealed distinct infection patterns. High-content screening analysis showed that in SUMO1-KO cells, SARS-CoV-2 exhibited single-cell infection with a higher infection rate, whereas clustered infection and increased cell fusion were observed in SUMO2-KO cells compared to wild-type (WT) A549-hACE2 cells (Figure 1B). Strikingly, quantitative PCR analysis showed that SUMO1 knockout dramatically enhanced production of virion particles in the supernatant, but remained no significantly change of viral RNA copies within cells. In contrast, SUMO2 knockout enhanced viral RNA levels within the cells with no impact on virion production (Figure 1C-D). These findings highlight the different role of SUMO1 and SUMO2 signaling in SARS-CoV-2 infection: SUMO1 restrains viral particle release into the supernatants but promotes cell fusion, whereas SUMO2 suppresses cell fusion and enhances viral replication within cells.

### SARS-CoV-2 Spike Protein Undergoes SUMO2 Modification and Contributes to Perinuclear Punctate Distribution

**Figure 2:**
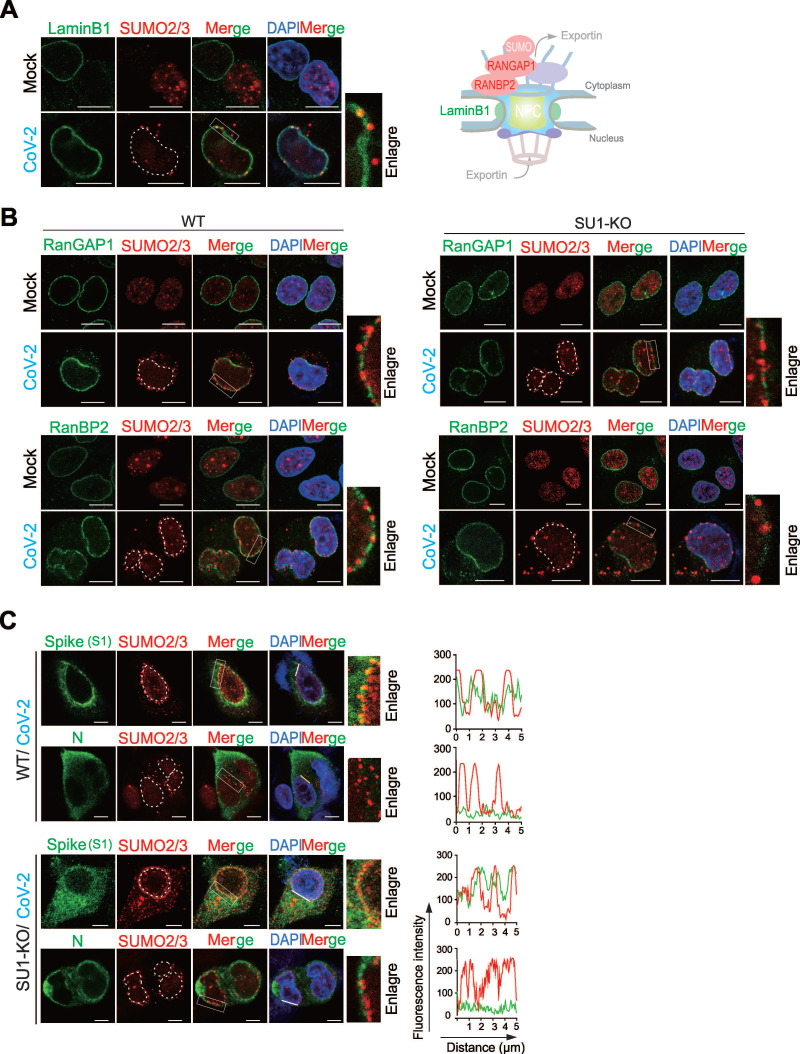
**Identification of host and viral proteins related with perinuclear punctate distribution of SUMO2/3 induced by SARS-CoV-2 infection.** (**A**) Perinuclear punctate distribution of SUMO2/3 induced by CoV-2 infection co-localize with Lamin B1. A549-hACE2 cells, uninfected (Mock) or infected with CoV-2 (strain SH01, MOI = 1) for 24 h, were analyzed by immunofluorescence using the antibodies as indicated in the figure. A schematic of the key molecules in nuclear pore complex represent in right panel. Scale bar, 10 μm. (**B**) Perinuclear punctate distribution of SUMO2/3 induced by CoV-2 infection does not co-localize with RanGAP1 and RanBP2. wild-type A549-hACE2 cells or with knockout of SUMO1 (SU1-KO), either uninfected (Mock) or infected with CoV-2 (strain SH01, MOI = 1) for 24 h, were analyzed by immunofluorescence using the antibodies as indicated in the figure. Scale bar, 10 μm. (**C**) Co-localization of Spike not N protein with SUMO2/3 in perinuclear puncta. A549-hACE2 cells, either wild-type or with knockout of SUMO1 (SU1-KO), were subjected to CoV-2 infection as in panel B, followed by immunofluorescence assays using the antibodies as indicted in the figure. The white line marks the position analyzed for co-localization signals, as shown in the right panel. Nuclei were stained with DAPI, and the scale bar presents 5 μm.

In the view of the fact that the perinuclear punctate distribution of SUMO2/3 has not been reported in other viral infections, we speculated that it may be important for SARS-CoV-2 infection. To explore which host molecule is induced by CoV-2 infection to localize with SUMO2/3 at perinuclear punctate region. We first tested the distributions of LaminB1 (a marker for the nuclear lamina), RanBP2 (a nucleoporin and an E3 SUMO protein ligase that has been reported previously in response to CoV-2 infection 30), and RanGAP1 (it typically forms a complex with SUMO-modified RanGAP1 and Ubc9 to co-regulate the SUMOylation of target proteins 31) of the nuclear pore complex upon viral infection (Figure 2A-B). The results showed that post-infection, SUMO2/3 partially co-localized with LaminB1 (Figure 2A), but not with RanGAP1 and RanBP2 (Figure 2B). Instead, the perinuclear punctate aggregation of SUMO2/3 is more frequently observed in the gaps of RanBP2 and RanGAP1 (Figure 2B). These results indicate that the perinuclear punctate aggregation of SUMO2/3 caused by CoV-2 infection does not occur at the nuclear pore complex.

Given that SARS-CoV-2 proteins are actively synthesized in the perinuclear endoplasmic reticulum (ER), which is located in close proximity to the SUMO2/3 perinuclear punctate region 32, 33. We hypothesized that viral proteins synthesized within the perinuclear ER may be SUMOylated, leading to the SUMO2/3 perinuclear puncta. To test this hypothesis, we first used GPS-SUMO prediction tool for whole genomic analysis of SARS-CoV-2 with potential SUMO modification, and identified that the Spike and N proteins were top 2 molecules with high score (Figure S2A). In the immunofluorescent assays of wild-type of A549-hACE2 and its derived SUMO1-KO cells with CoV-2 infection, we found that the CoV-2 Spike protein exhibited significant co-localization with SUMO2/3 in the perinuclear region, particularly in syncytial cells. In contrast, CoV-2 N protein displayed homogeneous cytoplasmic distribution without apparent co-localization (Figure 2C). These results suggest that the CoV-2 Spike is the key to induce SUMO2/3 perinuclear punctate distribution.

**Figure 3:**
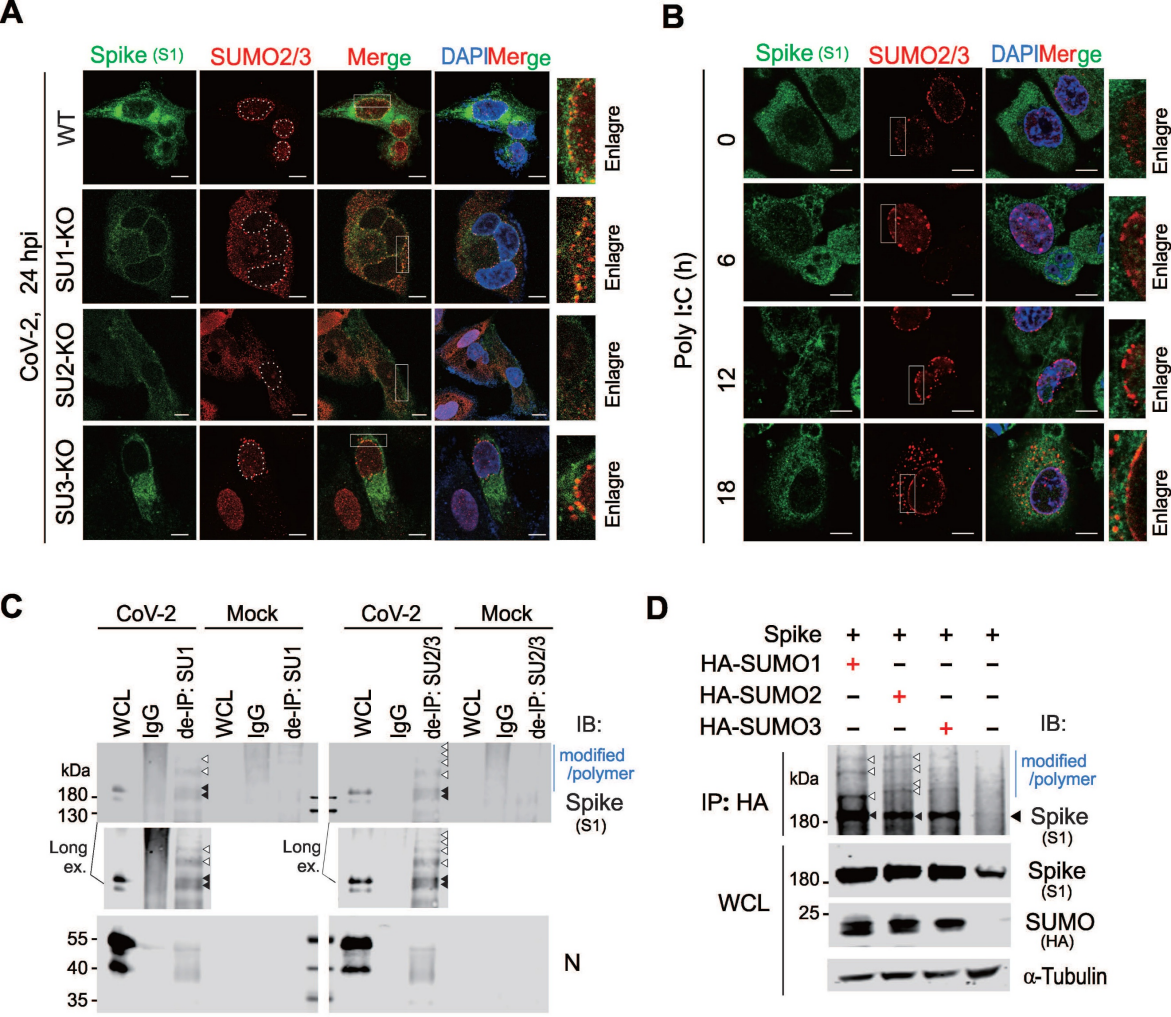
**SARS-CoV-2 Spike protein undergoes SUMO2 modification and dominantly localizes as perinuclear puncta.** (**A**) Knockout of SUMO2 abolishes dominant perinuclear punctate localization of Spike protein. A549-hACE2 cells, either wild type (WT) or with knockout of SUMO1(SU1-KO), SUMO2 (SU2-KO) or SUMO3 (SU3-KO), were infected with CoV-2 (MOI = 1) for 24 h, followed by immunofluorescence assays with antibodies as indicated in the figure. Co-localization of the Spike protein with endogenous SUMO2/3 is highlighted by the white box and enlarged in the right panel. Nuclei were stained with DAPI. The scale bar represents 5 μm. (**B**) Overexpression of Spike protein enhances SUMO2/3 perinuclear localization as puncta. A549-hACE2 SU1-KO cells were transfected with the Spike protein and treated Poly I:C at various time points starting 24 h post-transfection, followed by immunofluorescence using antibodies against Spike and SUMO2/3. The localization of the Spike protein and endogenous SUMO2/3 is highlighted by the white box and enlarged in the right panel. The scale bar represents 5 μm. (**C**) Analysis of endogenous Spike protein SUMOylation. Whole cell lysates (WCL) from A549-hACE2 cells, either uninfected (Mock) or infected with CoV-2 (SH01, MOI = 1) for 24 h, were subjected to denatured immunoprecipitation (de-IP) using antibodies against endogenous SUMO1 (SU1) and SUMO2/3 (SU2/3), followed by immunoblotting (IB) with antibodies as indicated in the figure. Black arrows indicate native bands of the Spike protein, while white arrows point to modified and polymerized forms of Spike. The CoV-2 N protein served as an internal control. (**D**) Analysis of SUMOylation of exogenous CoV-2 Spike protein. HEK293T cells were transfected with plasmids encoding Spike protein along with HA-tagged SUMO1, SUMO2, or SUMO3. At 48 h post-transfection, whole cell lysates (WCL) were subjected to immunoprecipitation (IP) using an HA-tag antibody, followed by immunoblotting (IB) with the antibodies indicated in the figure.

To determine it is SUMO2 or SUMO3 to undergo perinuclear punctate distribution, we infected A549-hACE2 and corresponding SUMO deficient cell lines with SARS-CoV-2. Notably, no SUMO-related perinuclear punctate distribution appeared in A549-hACE2 SUMO2-KO cells, and the distribution of CoV-2 Spike in the endoplasmic reticulum was greatly reduced compared to other groups (Figure 3A). To further confirm how the Spike protein influences SUMO2 perinuclear punctate distribution, we overexpressed CoV-2 Spike in A549-hACE2 SUMO1-KO cells and stimulated the cells with Poly I:C for 18 h post-transfection to mimic RNA virus infection process. Interestingly, without Poly I:C stimulation, CoV-2 Spike alone slightly induce SUMO2 perinuclear punctate distribution, which was significantly enhanced upon prolonged Poly I:C stimulation, peaking at 18 h (Figure 3B). This specificity highlights the unique role of the Spike protein in modulating SUMO2 redistribution in the perinuclear region. To rule out interference from other viral proteins, we tested the effects of Poly I:C stimulation alone, as well as the overexpression of other viral proteins such as the N protein and ORF6, known to interact with the nuclear pore complex 29. None of these interventions influenced the intranuclear localization of SUMO2/3 (Figure S2B-C), further confirming the specificity of the Spike protein's effect.

To investigate the potential mechanism underlying SUMO2 perinuclear punctate distribution by Spike, we disrupted non-covalent linkage between proteins using denatured immunoprecipitation. This method showed that Spike could be captured by antibodies against SUMO1 and SUMO2/3, suggesting that it undergoes SUMO modification (Figure 3C). To validate our approach, we examined the N protein, known to be SUMOylated, and found that it was effectively immunoprecipitated by SUMO1 and SUMO2/3 antibodies, similar to the Spike protein (Figure 3C). Additionally, we co-expressed CoV-2 Spike with HA-tagged SUMO1, SUMO2, and SUMO3 in HEK293T cells (Figure 3D), and performed co-immunoprecipitation assays. The results showed that Spike interacted with all three SUMO isoforms, and SUMO overexpression increased the expression level of Spike (Figure 3D). These findings suggest that the SUMO2 perinuclear puncta observed in SARS-CoV-2-infected cells are primarily associated with SUMO-modified Spike.

### SUMO2 Modification Suppresses Viral Cell-to-Cell Transmission by Inhibiting Spike Proteolytic Cleavage

**Figure 4:**
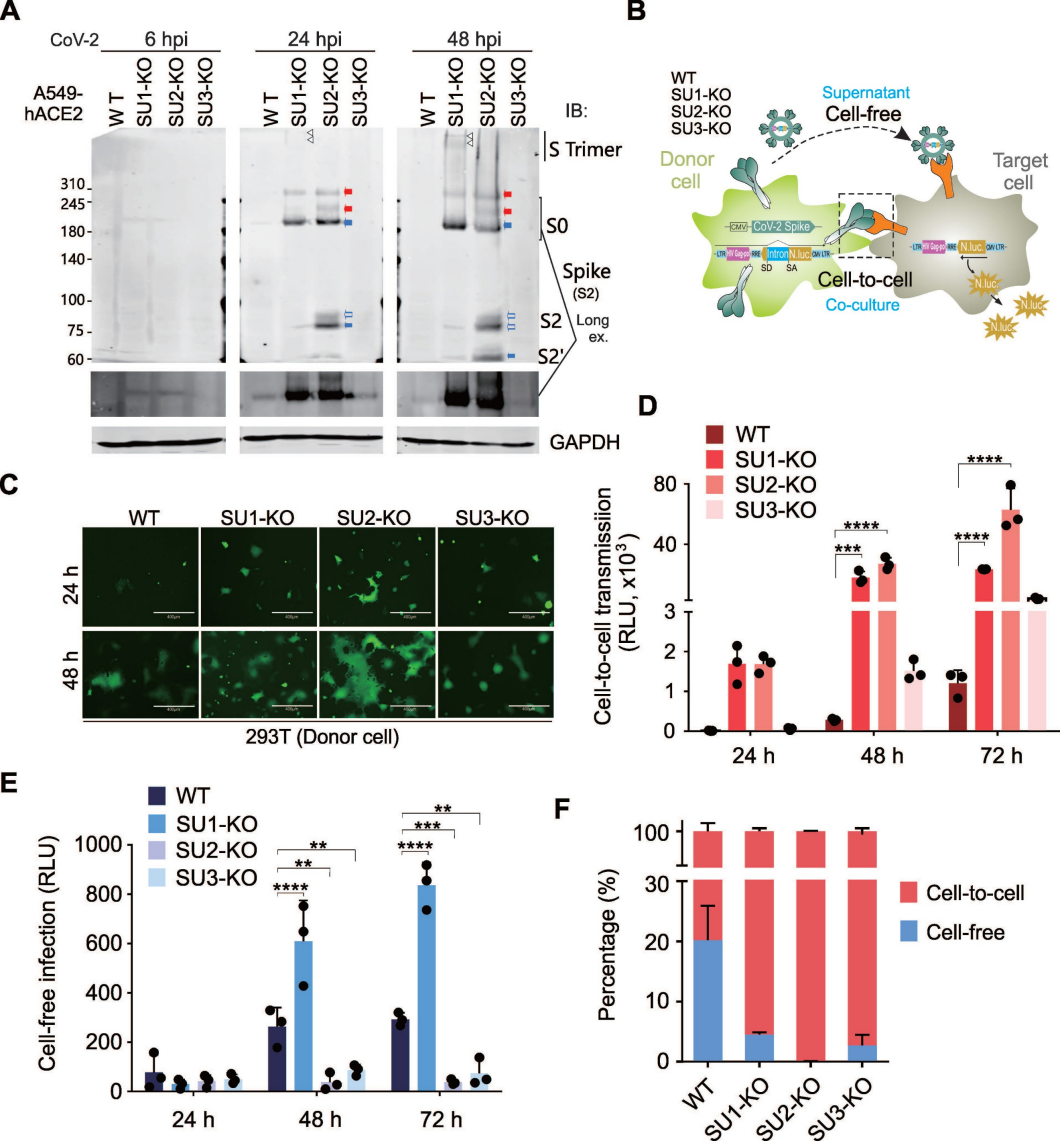
** SUMO2 KO in donor cells enhances Spike protein cleavage and viral cell-to-cell transmission.** (**A**) SUMO2 KO in donor cells increases Spike protein cleavage and modified forms. Cell lysates from A549-hACE2 cells infected with CoV-2 (SH01, MOI = 1) at various time points were analyzed by immunoblotting (IB) using antibodies against Spike (S2). Blue arrows indicate the ~190 kDa band corresponding to the native Spike (S0) protein, while red arrows highlight modified bands. Empty blue arrows point to the cleaved fragments of S2 and S2'. Triangle indicates the trimeric form. (**B**) Schematic representation of SUMO modification's effect of Spike protein on cell-to-cell transmission. Donor cells, either wild-type (WT) or SUMO KO (SU1-KO, SU2-KO, SU3-KO) HEK293T cells, were co-transfected with plasmids encoding the Spike protein, an NL4.3-inNluc reporter, and eGFP. At 24 h post-transfection, donor cells were co-cultured with target cells A549-hACE2 for 72 h, after which luciferase activity was measured. For cell-free infection, virus was harvested from an equivalent number of transfected donor cells and used to infect equal numbers of A549-hACE2 cells and untransfected donor cells. (**C**) SUMO2 KO enhances Spike-mediated syncytia formation. Immunofluorescence analysis of donor cells at 24 h and 48 h post-co-culture with target cells (as described in panel **B** revealed syncytia formation in SUMO2 KO cells. Scale bar, 10 μm. (**D**) SUMO2 KO dominantly enhances Spike-mediated cell-to-cell transmission. Data is from three independent experiments in panel **B**. (**E**) SUMO1 KO exclusively induces Spike-mediated cell-free infection. Three independent experiments in panel **B** show that SUMO1 KO specifically enhances cell-free infection mediated by the Spike protein. (**F**) SUMO2 KO dominantly increases the ratio of cell-to-cell to cell-free infection mediated by the Spike protein. The relative ratio of cell-to-cell to cell-free infection was calculated from data in panels **D** and **E**. Each bar represents the mean ± standard deviation. **p < 0.01; *** p < 0.001; and **** *p* < 0.0001.

To explore the impact of SUMOylation on the CoV-2 Spike protein, we conducted immunoblotting experiments to evaluate Spike protein expression in A549-hACE2 cells and SUMO-KO cell lines following SARS-CoV-2 infection. The data revealed that the depletion of SUMO2 results in an increase presence of the cleaved form of Spike protein and a decrease in its trimeric configuration. Conversely, the absence of SUMO1 led to an accumulation of the trimeric form and a reduction in the cleaved products (Figure 4A). Given that SARS-CoV-2 can spread through both free-floating virions and direct cell-to-cell contact 29, and Spike protein cleavage facilitates the later 34, we utilized an HIV-1 lentiviral vector carrying an intron-luciferase (InNluc) to construct a system for detecting the cell-to-cell transmission capability of the Spike protein. This system comprises donor cells packaging Spike-pseudotyped viruses and target cells expressing luciferase.

We individually knocked out SUMO1, SUMO2, or SUMO3 in donor cells (293T) (Figure 4B and Figure S3A-B) or target cells (A549-hACE2) (Figure S4A), to determine the impact of SUMOylation on Spike function during protein synthesis or viral infection. Consistently, fluorescence microscopy showed that SUMO2 KO in donor cells significantly increased Spike-mediated cell fusion (Figure 4C). Luciferase reporter assays further confirmed that donor cell significantly enhanced the cell-to-cell transmission while diminishing cell-free infection ability. In contrast, SUMO1 KO in donor cells had the opposite effect, increasing both modes of transmission (Figure 4D-E). Notably, nearly all (99%) CoV-2 Spike pseudoviruses produced in SUMO2-KO donor cells were transmitted via cell-to-cell contact (Figure 4F), mirroring the similar pattern observed in CoV-2-infected SUMO2-KO cells (Figure 1B). Consistence with the observation in donor cells with SUMO deficiency, the impact of SUMO depletion on Spike protein function in target cells is totally opposite. Knockout of SUMO1 and SUMO2 in target cells significantly reduces cell-to-cell transmission capacity (Figure S4B-E). These observations suggest that SUMO2 modification of Spike inhibits cell-to-cell transmission by preventing its proteolytic cleavage.

### SUMOylation at K129 and K1269 Regulates Spike Protein Cleavage

**Figure 5:**
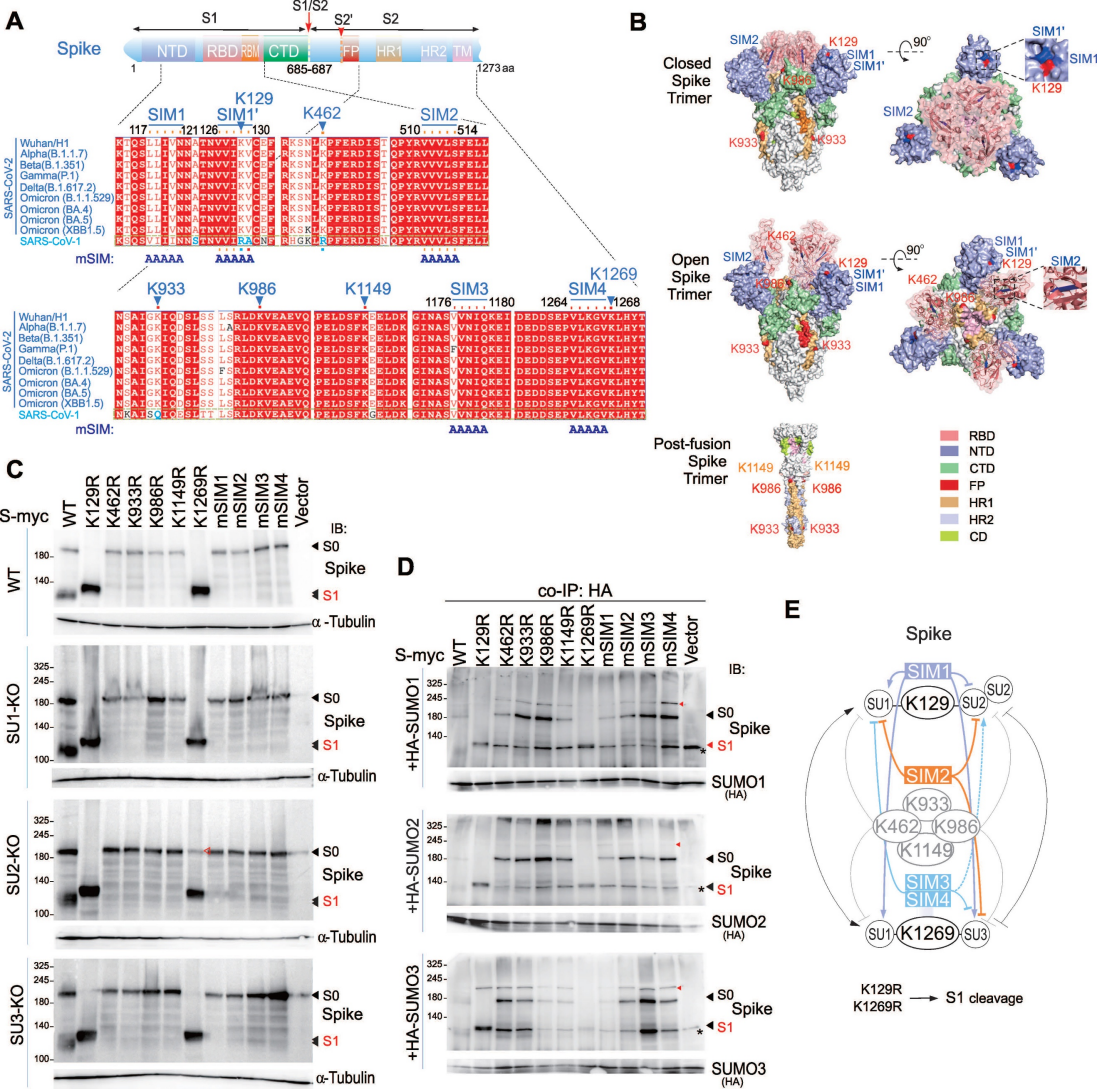
**Identification of SUMOylated sites and SIM motifs in the Spike protein.** (**A**) Schematic representation of the Spike protein highlights its SUMOylated sites and SUMO-interacting motifs (SIM). Sequence alignments of these sites from various CoV-2 variants and CoV-1 were predicted using GPS-SUMO software. The identical positions are shown in red, similar positions in white, and amino acids differing between CoV-1 and CoV-2 are highlighted in blue. The mutation of the SIM motif (mSIM) is indicated. (**B**) Three-dimensional spatial distribution of SUMOylated sites and SIM motifs of Spike protein. The three-dimensional locations of SUMOylated sites and SIM motifs on the atomic models of the closed (PDB ID 6VXX) and open (PDB ID 7X7N) forms of the Spike trimer are depicted in side and top views. SUMOylated sites are marked in read, and SIM motifs in blue. Notably, K1269 and SIM3/4, located in the transmembrane and cytoplasmic regions, are not visible in the figure. The bottom panel shows the locations of K1149, K986 and K933 in the post-fusion form of Spike trimer (PDB ID 6XRA). (**C**) Impact of mutation of SUMOylated sites and SIM motifs on Spike protein cleavage. Wild-type (WT) HEK293T cells and derived cell lines with knockout of SUMO1(SU1-KO), SUMO2 (SU2-KO) or SUMO3 (SU3-KO) were transfected with myc-tagged WT Spike and its mutants. At 48 h post-transfection, cell lysates were analyzed by immunoblotting (IB) assays using antibodies against Spike (600-700). The bands corresponding to S1 cleavage products are indicated. α-tubulin served as an internal control. (**D**) Effect of mutations on SUMOylation of the Spike protein. HEK293T cells were transfected with myc-tagged WT Spike or its mutants, either in the presence or absence of HA-tagged SUMO. At 36 h post-transfection, cells were treated with MG132 for 12 h before being harvested for co-immunoprecipitation (co-IP) using HA antibodies, followed by immunoblotting (IB) with antibodies against Spike (600-700). Alternatively, directly immunoblotting was performed and shown in supplementary Figure S4B. Red arrows indicate the SUMO-modified forms of the Spike protein, while an asterisk denotes a non-specific band. (**E**) A schematic diagram illustrates the effects of mutations in SUMOylated sites and SIM motifs on SUMO modification of Spike protein, as analyzed through artificial intelligence in panel **C** and **D**. The role of lysine residue mutations at positions 129 and 1269 is highlighted in the bottom panel.

To further identify specific SUMOylation sites on the Spike protein, we used the GPS-SUMO prediction tool to analyze the Spike sequence, and identified several potential SUMOylation sites and SUMO-interacting motifs (SIMs) that match the consensus sequence V/I/L-X-V/I/L-V/I/L motif (Supplementary Figure S5A). Among these, two SIM motifs, designated SIM1 and SIM1', are located in close proximity (Figure 5A). Comparing the conservation of these sites between SARS-CoV-1 and SARS-CoV-2 variants of concern (VOCs), we found high conservation of SUMOylation sites and SIMs in SARS-CoV-2 variants, even in the highly variable N-terminal domain (NTD) and receptor-binding domain (RBD) of S1 (Figure 5A). However, these sites are not uniformly conserved between SARS-CoV-1 and SARS-CoV-2; specifically, positions 462 and 933 in SARS-CoV-1 do not correspond to lysine (K) residues, and the region linking SIM1 and SIM1' diverges from that in SARS-CoV-2. This suggests that SUMOylation sites and SIM on Spike have played a significant role in the evolution of SARS-CoV-2. Mapping these sites onto the three-dimensional structure of the Spike's extracellular domain in its closed, open, and post-fusion conformations, shows that all discernible SUMOylation sites are surface-exposed. The opening of the RBD further exposes sites 986 and 462, while SIM1 and SIM1' remain spatially close, and only SIM2 is located within inside part of the RBD domain (Figure 5B).

To examine the functional importance of these sites, we sequentially mutated the predicted SUMOylation sites by substituting lysine (K) with arginine (R) and the SIM residues with alanine (A), creating mutants named Spike mSIM1/2/3/4. We then transiently transfected these mutants into 293T wild-type (WT) and SUMO-KO cell lines. Immunoblotting analysis indicated that mutations at positions K129 and K1269 significantly enhanced Spike cleavage. In WT cells, both K129R and K1269R mutants were present in the cleaved S1/S2 form, whereas in SUMO2-KO cells, but not SUMO1-KO or SUMO3-KO cells, the K1269R mutant partially restored the original size of S0. In contrast, all other mutants inhibited Spike cleavage, remaining in the form of S0 (Figure 5C). Co-expression of these mutants with HA-tagged SUMO1, SUMO2, and SUMO3 in 293T cells followed by co-immunoprecipitation revealed that mutations at individual sites did not completely block Spike SUMOylation, but some sites showed a preference for specific SUMO modifications. For example, K462R reduced SUMO1 modification, and mSIM1 decreased both SUMO1 and SUMO3 modifications (Figure 5D and supplementary Figure S5B). The Spike protein displayed a diverse pattern of SUMO modifications, as detail shown in Figure 5E. Specifically, SIM1 promotes SUMO1 modifications, while SIM3/4 promote SUMO2 modifications on lysine residues 129 and 1269, respectively. Importantly, these modifications can be efficiently disrupted by the SIM2 motif. These results demonstrate that SUMOylation sites and SIMs on the Spike protein regulate its cleavage and suggest potential antiviral mechanisms.

### The SIM2-derived Peptide cpSIM2 Inhibits SARS-CoV-2 Infection and Cell Fusion

**Figure 6:**
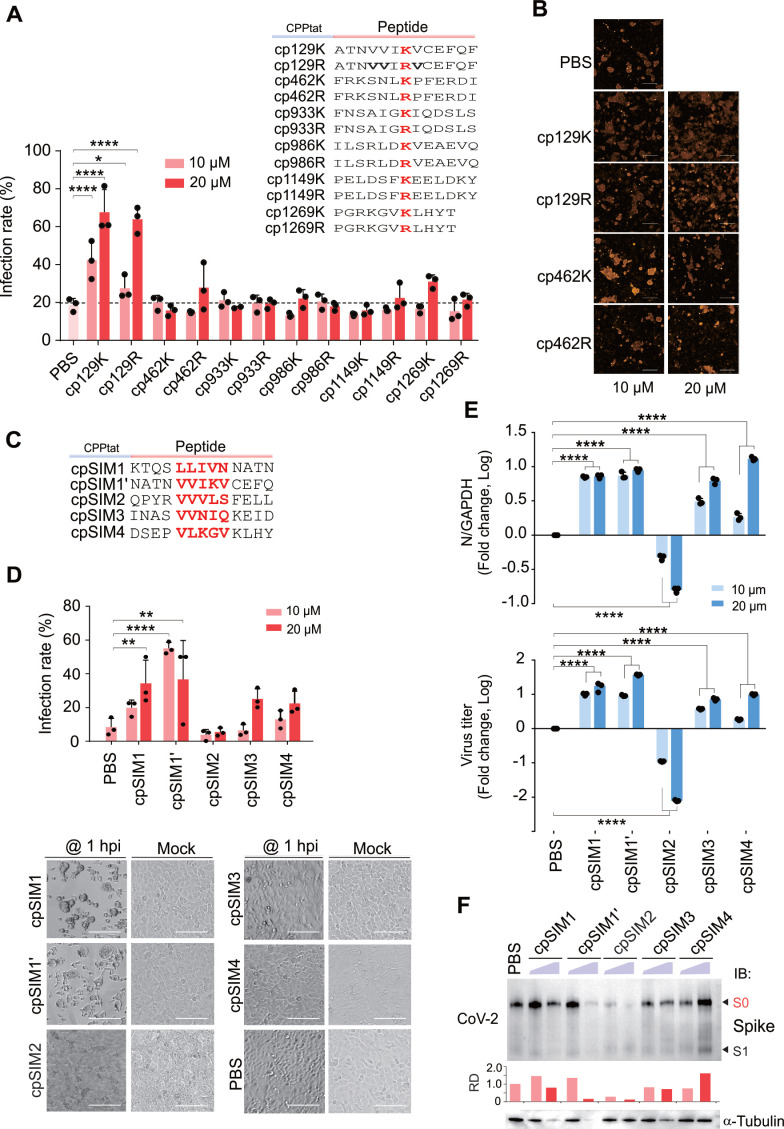
**The cpSIM2 peptide derived from the Spike protein inhibits SARS-CoV-2 replication.** (**A**) Effect of SUMOylated site-derived peptides on CoV-2 replication. A549-hACE2 cells, either uninfected (Mock) or infected with CoV-2 (strain SH01, MOI = 1), were treated with the indicated peptides or PBS for 18 h starting 2 h post-infection. The infection rates of cells treated with the different dosage of peptides were determined by high-content scanning microscopy. Schematic of peptides derived from the SUMOylated sites of the Spike protein are shown in the top panel. Peptides derived from the SUMOylated sites of the Spike protein, including cp129K/R, cp462K/R, cp933K/R, cp986K/R, cp1149K/R, and cp1269K/R, were synthesized with a cell-penetrating peptide (CPPtat) fusion. The lysine residues are highlighted in red. (**B**) Representative images of cell morphology assessed using high-content scanning microscopy in panel **A**. Scale bar, 20 μm. (**C**) Schematic of peptides derived from the SIM motifs of the Spike protein. Peptides derived from the SIM motifs of the Spike protein, including cpSIM1, cpSIM1', cpSIM2, cpSIM3, and cpSIM4, were synthesized with a cell-penetrating peptide (CPPtat) fusion. The SIM motifs are highlighted in red. (**D**) The infection rates of cells treated with the peptides were determined by high-content scanning microscopy. A549-hACE2 cells, either uninfected (Mock) or infected with CoV-2 (strain SH01, MOI = 1), were treated with the indicated peptides or PBS for 18 h starting 1 h post-infection. Lower panel, representative images of SIM-derived peptides on cell morphology with or without viral infection. Scale bar, 40 μm. (**E**) Effect of SIM-derived peptides on CoV-2 replication. The relative viral replication within host cells (upper panel) and virion particle production in supernatants (lower panel) from panel D were analyzed by qPCR targeting the CoV-2 N gene. All analyses were conducted using the same A549-hACE2 cells as in panel **D**. **p < 0.01; *** *p* < 0.001; and **** *p* < 0.0001. (**F**) Immunoblotting was performed on cell lysates from panel b using antibodies against the Spike protein (600-700). The relative density (RD) of the native Spike (S0) band was quantified and is shown in the middle panel. The α-tubulin served as an internal control.

To determine the role of blocking SUMOylation of the CoV-2 Spike protein on viral infection, we designed and synthesized a series of interfering peptides based on the SUMOylation sites of the Spike, conjugated to a cell-penetrating peptide (CPP) to facilitate energy-independent cellular uptake (Figure 6A). Treatment of A549-hACE2 cells infected with SARS-CoV-2 (SH01) with these peptides revealed that cp129K and cp129R increased the rate of viral infection and cell fusion, while cp1269K and other interfering peptides had no significant effect on infection rates (Figure 6A-B). Quantitative PCR analysis showed that cp129K and cp129R did not significantly alter the levels of CoV-2 N and E genes, indicating that the enhancement of viral infection was not due to increased viral replication (Figure S6A). Immunoblotting analysis confirmed that cp129K and cp1269K treatments increased the amount of cleaved S1/S2 form of the Spike protein (Figure S6B), demonstrating that cp129K promotes SARS-CoV-2 infection and cell fusion by facilitating Spike cleavage.

Considering that the SIM1' motif is present in both cp129K and cp129R, we hypothesized that the SIM of Spike protein might play a more substantial role in SARS-CoV-2 infection. Therefore, we designed and synthesized multiple interfering peptides targeting the SIM motifs of the Spike protein (Figure 6C). Treatment of A549-hACE2 cells infected with SARS-CoV-2 (SH01) with these peptides for 18 h, revealed that cpSIM1, cpSIM1', cpSIM3, and cpSIM4 all increased the SARS-CoV-2 infection rate in a dose-dependent manner (Figure 6D). Microscopic observations indicated that these four peptide treatments facilitated the formation of numerous syncytia and cell detachment, confirming that this effect was not due to drug toxicity (Figure 6D). In contrast, cpSIM2 inhibited SARS-CoV-2 infection in a dose-dependent manner (Figure 6D). Quantitative PCR results showed that cpSIM2 treatment suppressed both intracellular replication and supernatant virus release, with 20 μM cpSIM2 reducing supernatant virus release by 100-fold (Figure 6E). Immunoblotting confirmed that cpSIM2 treatment significantly reduced the level of Spike protein (Figure 6F).

### CpSIM2 Peptide Exhibits Broad-Spectrum Inhibitory Activity by Inhibiting Spike SUMO Modification

**Figure 7:**
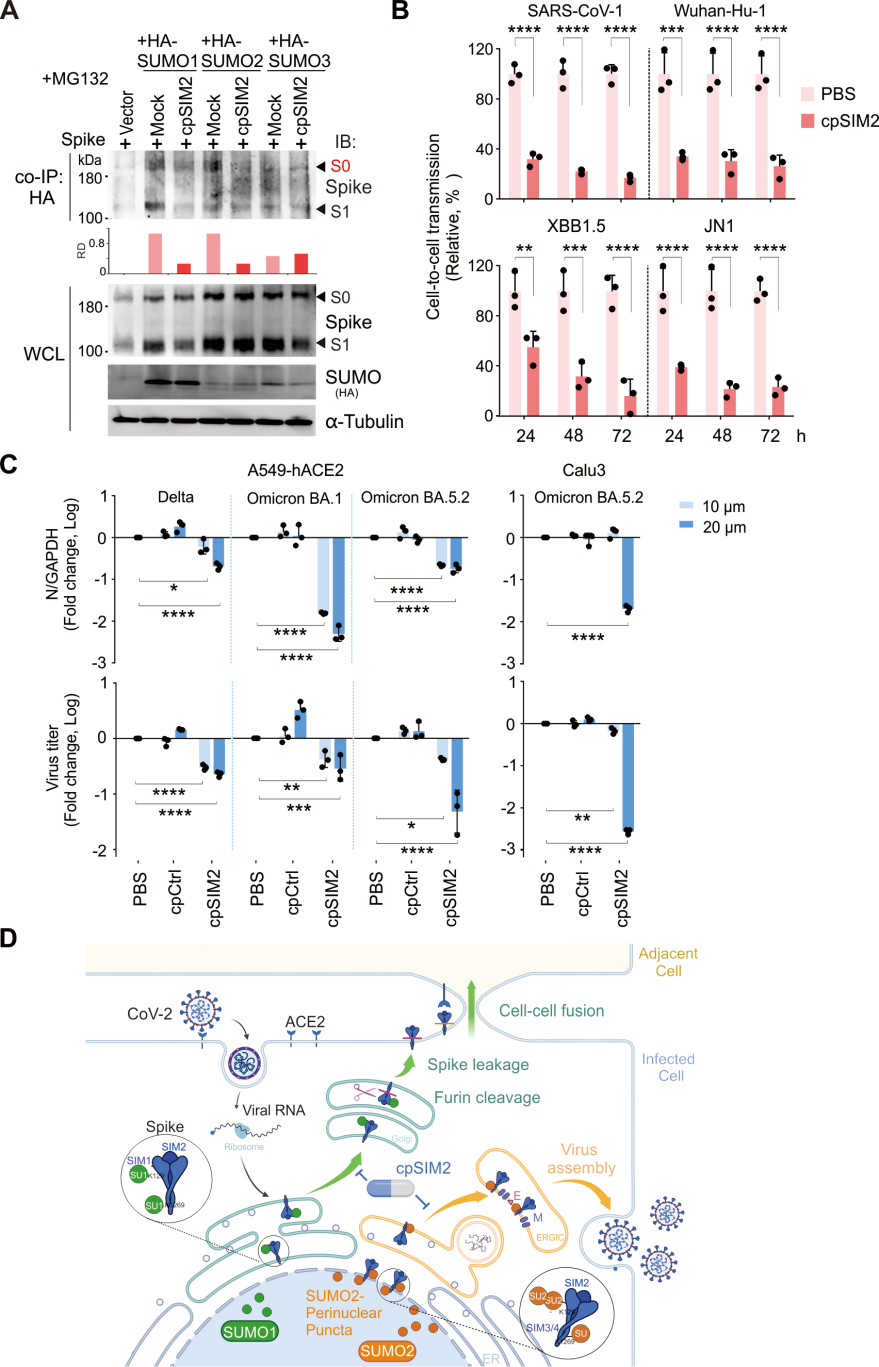
**The cpSIM2 peptide derived from the SIM2 motif of Spike protein efficiently inhibits replication of SARS-CoV-1 and SARS-CoV-2 variants *in vitro*.** (**A**) cpSIM2 peptide inhibits Spike SUMO modification. HEK293T cells were transfected with plasmids expressing Spike protein along with HA-tagged SUMO1, SUMO2, or SUMO3, followed by untreated (Mock) or treated with 10 μM cpSIM2. At 36 h post-transfection, cells were treated with MG132 for 12 h before harvest for co-immunoprecipitation (co-IP) and immunoblotting (IB) as indicated antibodies. The relative density (RD) of the native Spike form (S0) band was quantified and shown in the middle panel. (**B**) cpSIM2 blocks cell-to-cell transmission of SARS-CoV-1 and SARS-CoV-2 variant pseudoviruses. HEK293T donor cells were co-transfected with plasmids encoding the Spike protein of SARS-CoV-1 or SARS-CoV-2 variants and NL4.3-inNluc reporter, and treated with or without cpSIM2 (10μM). At 24 h post-transfection, donor cells were co-cultured with target cells A549-hACE2 for 72 h, after which luciferase activity was measured. Data are from three independent experiments. (**C**) cpSIM2 peptide inhibits replication of CoV-2 VOC strains *in vitro*. A549-hACE2 cells were individually infected with CoV-2 Delta, Omicron BA.1, and BA.5.2 strains (MOI = 0.1), and Calu3 cells were infected with CoV-2 Omicron BA.5.2 strain (MOI = 0.1). At 2 h post-infection, the infected cells were either untreated (PBS) or treated with cpSIM2 or a control peptide (cpCtrl) for 18 h. The relative viral replication within host cells (upper panel) and virion particle production in supernatants (lower panel) were analyzed by qPCR targeting the CoV-2 N gene. Data represents three independent replicates (n = 3). Each bar indicates the mean ± standard deviation. *p < 0.05; **p < 0.01; *** *p* < 0.001; and ****p < 0.0001. (**D**) Schematic representation of the dual role of SUMO modification on Spike-mediated cell-to-cell transmission and virion particle release. The SIM1-mediated SUMO1 modification of the Spike protein induces cleavage and facilitates cell-to-cell transmission, while the SIM3/4-mediated SUMO2 modification facilitates Spike protein localization at perinuclear puncta and promotes viral assembly and production. Notably, the SIM2 motif overrides the effects of both SIM1 and SIM3/4 motif-mediated SUMOylation of the Spike protein. The SIM2-derived cpSIM2 peptides efficiently blocks the expression of Spike protein, leading to the inhibition of CoV-2 viral replication. Created with BioRender.com (https://BioRender.com/0ybqss9).

To investigate the impact of cpSIM2 on CoV-2 Spike SUMOylation, we co-expressed CoV-2 Spike with HA-tagged SUMO1, SUMO2, and SUMO3 in HEK293T cells, followed by treatment with or without cpSIM2, and then performed co-immunoprecipitation assays. The results demonstrated that cpSIM2 significantly reduced the interaction levels between Spike and SUMO1/SUMO2, while its effect on SUMO3 was relatively minor (Figure 7A), indicating that cpSIM2 inhibits CoV-2 replication by blocking Spike SUMOylation.

Given the high conservation of SIM2 across SARS-CoV-2 variants, we assessed the broad-spectrum efficacy of cpSIM2 using the packaging Spike-mediated pseudovirus system described earlier (Figure 4A). Wild-type HEK293T donor cells were co-transfected with plasmids encoding the Spike proteins from SARS-CoV-1 or SARS-CoV-2 Wuhan-Hu-1, XBB 1.5, and JN.1, along with the NL4.3-inNluc reporter, and treated with or without cpSIM2. At 24 h post-transfection, donor cells were co-cultured with target A549-hACE2 cells to evaluate the effect of cpSIM2 on cell-to-cell transmission of these Spike-mediated pseudoviruses. The results showed that cpSIM2 peptide treatment significantly inhibited the cell-to-cell transmission ability of the pseudovirus bearing the SARS-CoV-1 and SARS-CoV-2 Wuhan-Hu-1, XBB 1.5, and JN.1 Spike proteins compared to the untreated group (Figure 7B).

Subsequently, we further evaluated the broad-spectrum efficacy of cpSIM2 using authentic viruses. A549-hACE2 cells were infected with SARS-CoV-2 Delta, Omicron variants BA.1, and BA.5.2, and then treated with cpSIM2. Consistent with the effects observed with the original strain, cpSIM2 treatment significantly inhibited intracellular replication and supernatant release of the SARS-CoV-2 variants. Additionally, treatment of SARS-CoV-2-infected Calu3 cells, a naturally infectious human lung cell line, also demonstrated significant antiviral effects (Figure 7C). Taken together, the results indicate that cpSIM2 could effectively block both SARS-CoV-2-induced cell-to-cell fusion and viral spread infection by disrupting the SUMOylation of the Spike protein (Figure 7D).

### CpSIM2 Blocks SARS-CoV-2 Replication* in vivo* with Favorable Safety Profile

**Figure 8:**
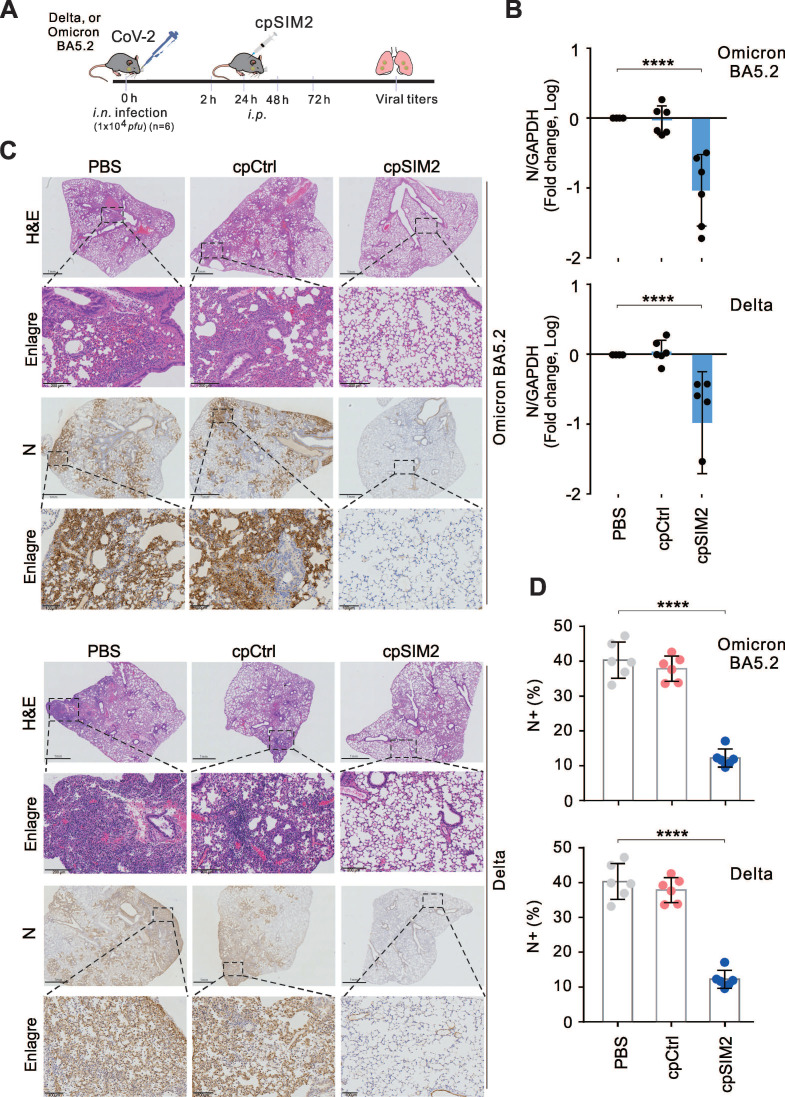
**The cpSIM2 peptide derived from the SIM2 motif of Spike protein efficiently inhibits replication of SARS-CoV-2 *in vivo*.** (**A**) Schematic diagram of mice with CoV-2 infection and cpSIM2 peptide treatment. C57BL/6-hACE2 mice (n = 6) were intranasally (*i.n.*) inoculated with CoV-2 (strain Delta or Omicron BA.5.2), followed by intraperitoneally (*i.p.*) administration of either cpCtrl (25 mg/kg), cpSIM2 (25 mg/kg), or PBS every 24 h for 4 days. On day 5 post-infection, the mice were euthanized, and lung tissues were collected for analysis. (**B**) The viral load in lung tissues from panel **A** was measured by quantitative PCR targeting the CoV-2 N gene. Each bar represents the mean ± standard deviation. ****p <0.0001, indicating statistically significantly differences. (**C**) Viral load in lung tissues was assessed by immunohistochemistry using antibodies against the CoV-2 N protein. Hematoxylin and eosin (H&E) staining was performed for pathological examination, and representative magnified images are shown in the lower panels. (**D**) The percentage of N-positive cells in the lung tissues from* panel C* was quantified using the ImageJ software.

To validate the antiviral effects of cpSIM2 *in vivo*, the hACE2 transgenic mice were intranasally inoculated with SARS-CoV-2 Delta or Omicron BA5.2, and treated intraperitoneally with PBS, cpControl (cpCtrl), or cpSIM2 at 2, 24, 48, and 72 h post-inoculation (Figure 8A). On the fifth day after infection, all mice were euthanized, and lung tissues were collected for viral quantitative PCR analysis. The results showed that cpSIM2 treatment significantly suppressed SARS-CoV-2 Delta and Omicron BA5.2 replication in the lungs compared to PBS-treated and cpCtrl-treated groups (Figure 8B). Hematoxylin and eosin staining indicated that SARS-CoV-2 infection caused severe lung damage in PBS-treated and cpCtrl-treated mice, which was mitigated by cpSIM2 treatment (Figure 8C). Immunohistochemical staining for the N protein revealed a remarkable reduction in virus distribution in the lungs of cpSIM2-treated mice (Figure 8D). The results demonstrate that the cpSIM2 peptide exhibits broad-spectrum anti-SARS-CoV-2 activity *in vivo*.

To preliminary evaluate the safety of cpSIM2* in vivo*, we conducted a toxicity test in mice with intraperitoneally administration of cpSIM2 at different dosage every day for 4 days. On day 5 post-treatment, mice were euthanized, and blood samples and organ tissues were collected for analysis (Figure S7A). The results showed that compared to PBS control group, three different dosages of cpSIM2 treatment did not significantly impair the levels of some key biochemical and hematological parameters, including alanine aminotransferase (ALT), aspartate amino-transferase (AST), and white blood cell (WBC) counts (Figure S7B and Table S3). In the histopathological analysis of major organs (heart, liver, spleen, lung, and kidney) with hematoxylin and eosin staining, we did not observe any significant damage of pathological tissues in the cpSIM2-treated mice (Figure S7C). These findings indicate that cpSIM2 is a potential safe therapeutic agent for further development as anti-CoV drugs.

## Discussion

Viruses have evolved a multiple of strategies to hijack host cellular mechanisms, including the regulation of post-translational modifications (PTMs), to suppress host antiviral responses and enhance their survival and replication efficiency 10. SUMOylation, a key PTMs, has been demonstrated to play critical roles in regulating both host and viral gene expression, as well as immune responses 35. Emerging evidence indicates that virus can encode specific proteins that interfere with the host's SUMOylation process, acting either as substrates or regulators for SUMO 11. In this study, we identified SUMOylation as a critical therapeutic target for SARS-CoV-2 infection by revealing its dual regulatory roles in Spike protein: SUMO1 modification induces Spike cleavage and enhances cell-to-cell transmission, whereas SUMO2 modification localizes the Spike protein to the perinuclear region, facilitating virion particle release. Building on this mechanism, we engineered cpSIM2, a cell-penetrating peptide derived from the conserved SIM2 motif, capable of concurrently inhibiting both SUMO1 and SUMO2 modifications of the Spike protein, thereby exerting potent antiviral activity. Crucially, cpSIM2 demonstrates potent broad-spectrum antiviral efficacy against SARS-CoV-1 and SARS-CoV-2 variants. The discovery of SUMO-modified Spike protein not only deepens our knowledge of how SARS-CoV-2 manipulates host PTMs but also paves the way for the development of broad-spectrum strategies against emerging coronavirus strains.

Although previous studies have shown that host SUMOylation plays a role in SARS-CoV-2 infection, including the SUMOylation of the ACE2 receptor and N protein 12, 14, our study identified for the first time the SUMOylation of the viral structural protein Spike and its heterogeneous regulation via different SUMO isoforms for viral infection. Consistence with previous observation that SUMO modification can mediate the redistribution of target proteins, and recent high-resolution imaging studies show that the SARS-CoV-2 Spike protein localizes to the nuclear membrane during late stages of infection 36, our findings suggest that this re-localization may be driven by SUMO modification and linked to the perinuclear puncta of SUMO2. In addition, consistent with previous report that SUMOylation of nuclear lamina proteins A/C can induce perinuclear punctate of SUMO1 in response to nuclear DNA leakage 37, we found co-localization of SUMO2/3 with the nuclear envelope protein LaminB1, indicating that LaminB1 may play a role in the SARS-CoV-2-induced perinuclear punctate distribution of SUMO2. Further investigation is required to elucidate the specific function of LaminB1 in this context.

Beyond SUMOylation, viral surface proteins commonly undergo glycosylation, which is essential for protein maturation and viral entry 38, 39. However, the SUMOylation of viral surface proteins and its functional implications have been less explored. Our research identifies the Spike protein as the first RNA viral surface membrane protein capable of undergoing SUMOylation, opening new avenues in RNA virus research. The effects of SUMOylation on other RNA viral proteins are diverse, for instance, SUMOylation of HIV-1 p6 and influenza virus (IAV) Non-structural protein 1 (NS1) inhibits viral replication 40, 41, whereas SUMOylation of IAV, SARS-CoV-1 and SARS-CoV-2 NP protein facilitates viral assembly and release 12, 42, 43. In this study, we discovered that SUMO2 modification inhibits CoV-2 Spike protein cleavage and cell-to-cell transmission, while SUMO1 has an opposing effect. We hypothesize that these discrepancies arise from distinct temporal dynamics of SUMO1 and SUMO2 modifications, which may have competing effects on the virus infection. The presence of multiple SUMOylation sites and SIM motifs with the Spike protein, along with the effects of interfering peptides, underscores the complexity of Spike SUMOylation and its role in viral replication and cell-to-cell transmission.

It is known that viruses can spread through two main pathways, including being packaged into virions for release and subsequent infection of new cells, and direct cell-to-cell transmission via cell fusion 19. For SARS-CoV-2, the Spike protein is also packaged into virions via the trans-Golgi network (pathway 1), or transported to the cell membrane surface to interact with ACE2 receptor on neighboring cells, causing cell fusion (pathway 2) 44-46. During pathway 2, Spike is more prone to protease cleavage 18. Our study shows that SUMOylation regulates Spike protein trafficking between these two pathways. SUMO1 knockout shifts the balance toward pathway 1, increasing virion release and reducing cell fusion. This is supported by the evidence that SUMO1 knockout boosts Spike trimer formation and S1/S2 cleavage efficiency, indicating SUMO may affect Spike's interaction with key proteins during transport.

Given the ability of SIM motif to bind SUMO molecules, the SIM-derived peptide cpSIM2 may potentially interact with SUMO1 and SUMO2, thereby inhibiting the effects of SUMO1 and SUMO2 modifications and reducing Spike protein abundance, offering it as a potential therapeutic agent against SARS-CoV-2 infection. It is worthy to mention that SUMOylation inhibitors like ML-792 and GA significantly reduce the infection rate of pseudo-coronaviruses in cells and inhibit SARS-CoV-2 replication* in vivo* 14, which is consistent with the trend observed with cpSIM2, indicating that cpSIM2 indeed suppresses viral replication by inhibiting SUMOylation. Notably, the SIM2 motif is highly conserved across SARS-CoV-2 variants and SARS-CoV-1, indicating its potential to induce broad-spectrum antiviral effects on emerging coronavirus strains by disrupting a conserved SUMOylation mechanism. Targeting the host-virus co-evolutionary interface (SUMO-Spike interaction) provides a strategy with higher specificity compared to traditional approaches focusing on single host factors. Conversely, cpSIM1 and cpSIM1', derived from the SIM1 and SIM1' motifs, respectively, potently promote SARS-CoV-2 cell-to-cell fusion and replication, suggesting potential application in developing COVID-19 vaccine adjuvants to enhance Spike expression and presentation on the cell surface. In addition, it has been shown that SUMO tags are efficiently used in some COVID-19 vaccines to improve Spike RBD expression and purification 47, and our finding provides additional insights into this strategy. The reason why only cpSIM2 presents significant antiviral effects, rather than other peptides, could be attributed to the unique property of the SIM2 motif, which is located within the interior region of three-dimensional structure of the RBD domain, indicating its exclusive inhibitory activity against viral replication. Ginkgolic acid, a specific inhibitor targeting SUMOylation and membrane fusion 48, 49, has been shown to block the interaction between the Spike protein and ACE2, inhibiting SARS-CoV-2 and its variants 50. Molecular docking studies indicate that Ginkgolic acid forms hydrogen bonds with the 514 site of the Spike protein, coinciding with the SIM2 site identified in our study 50. This suggests that Ginkgolic acid and cpSIM2 may exert similar inhibitory effects by suppressing Spike SUMOylation, further emphasizing the importance of SUMOylation in Spike function.

In addition to SUMOylation, the CoV-2 Spike protein undergoes various PTMs, including glycosylation, palmitoylation, methylation and ubiquitination 20-23. These PTMs modulate Spike conformation, increase its binding affinity for ACE2, and promote membrane fusion. There is a dynamic interplay between SUMOylation and ubiquitination, which can modulate protein function through competitive or cooperative targeting of shared recognition motifs 23, 51. Previous studies have identified ubiquitination at three specific sites on the Spike protein (K462, K986 and K1269), which overlap with the SUMOylation sites reported here 52-54. Notably, K462 and K986 are exposed following the transition of the RBD to an open conformation, highlighting their role in Spike conformational shifts. The K986R mutation suppresses Spike cleavage and viral replication, consistent with inhibition of ubiquitination 53. This suggests a synergistic inhibitory role for SUMOylation and ubiquitination at these sites, potentially constituting part of the host antiviral response. Given that only a few ubiquitination sites on the Spike protein have been characterized, further investigation into the balance between SUMOylation and ubiquitination at additional lysine residues is warranted.

In summary, our study elucidates the critical role of SARS-CoV-2 Spike SUMOylation in viral replication and cell-to-cell transmission. Specifically, the peptide drug cpSIM2, by targeting Spike SUMOylation, effectively inhibits different strains of SARS-CoV-2 replication and cell-to-cell transmission, offering promise as a broad-spectrum therapeutic agent against SARS-CoV-2 variants.

## Supplementary Material

Supplementary figures and tables.
