# Supplementary material for: SUMOylation of SARS-CoV-2 spike protein is a key target for broad-spectrum antiviral therapy
Source: Theranostics. 2025 May 25;15(13):6369–86. doi: 10.7150/thno.111256 (PMC12160036; doi:10.7150/thno.111256)
Supplement: Supplementary file 1 — Supplementary figures and tables. [file thnov15p6369s1.pdf]

## Supplemental Figures and Tables

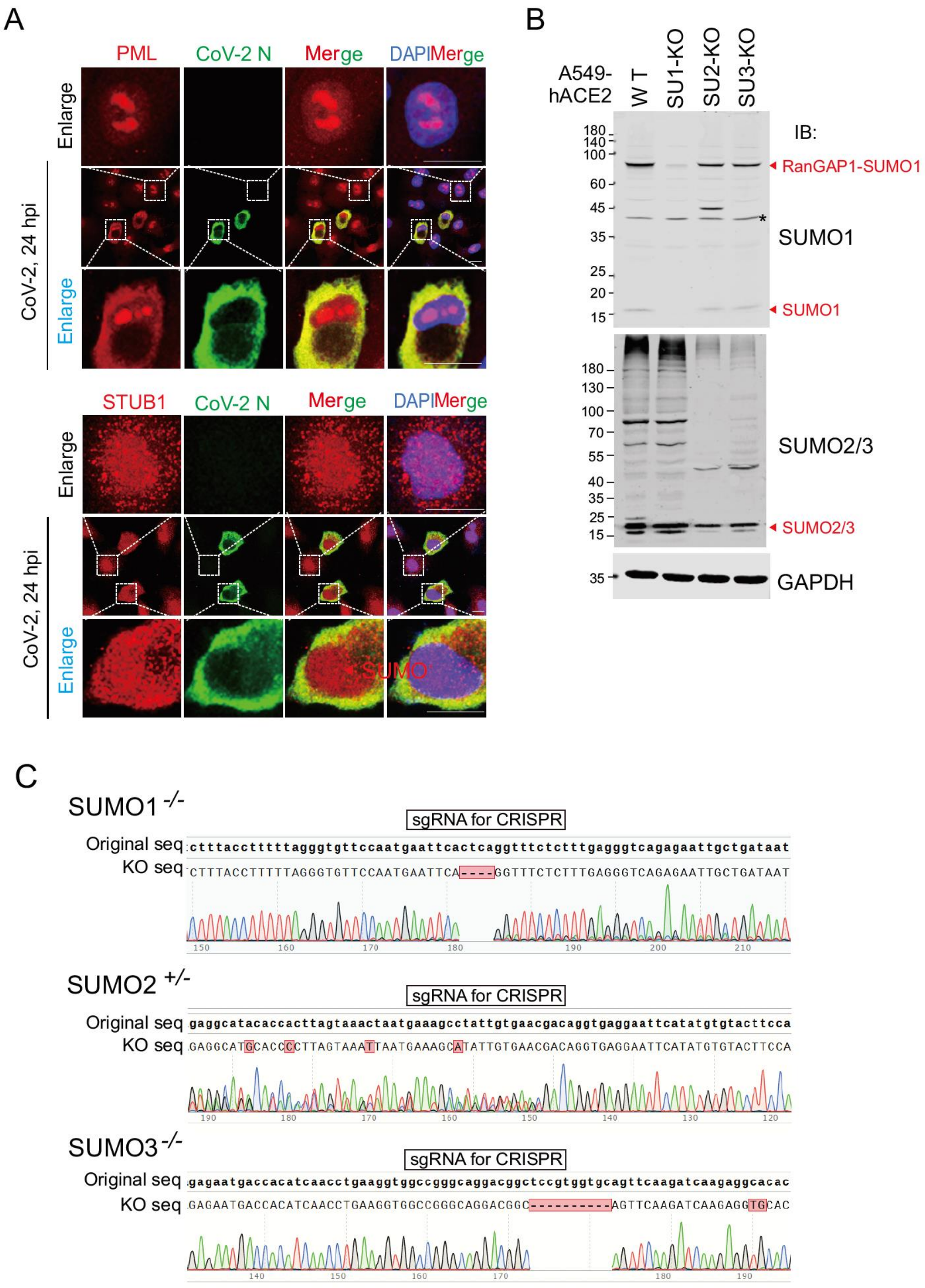


**Figure S1**. (**A**) Impact of SARS-CoV-2 infection on the subcellular localization of host SUMO-related proteins. A549-hACE2 cells were infected with CoV-2 (SH01,

MOI=1) for 24 h, followed by immunofluorescence analysis using the antibodies as indicated in figure. The scale bar represents 5 μm. Enlarged views of both infected and non-infected cells are shown, with nuclei stained with DAPI. (**B**) Expression level of SUMO protein in A549-hACE2 SUMO-KO cell lines. Whole cell lysates from wild-type (WT) or SUMO-knockout (SU1-KO, SU2-KO, and SU3-KO) A549-hACE2 cells were subjected to immunoblotting using the antibodies indicated in the figure. (**C**) Validation of SUMO1/2/3 gene knockout in the A549-hACE2SUMO-KO cell lines. Total DNA was extracted from A549-hACE2 SU1-KO, SU2-KO and SU3-KO cell lines, reverse-transcribed, and second-generation sequencing. The image shows the alignment of small guide RNA (sgRNA) targeting sequences for CRISPR-Cas9 with the original genomic sequences, confirming the successful knockout of the SUMO1/2/3 genes.

A

| ID | Position | Peptide | Score | Cut-off | P-Value | Type |
|---|---|---|---|---|---|---|
| S | 510 - 514 | VGYQPYR**VVVLS**FELLHAP | 44.466 | 29.92 | 0.03 | SUMO interaction |
| N | 130 - 134 | YGANKDG**IIWVA**TEGALNT | 44.416 | 29.92 | 0.036 | SUMO interaction |
| ORF1ab | 7086 - 7090 | IIRENNR**VVISS**DVLVNN* | 42.485 | 29.92 | 0.039 | SUMO interaction |

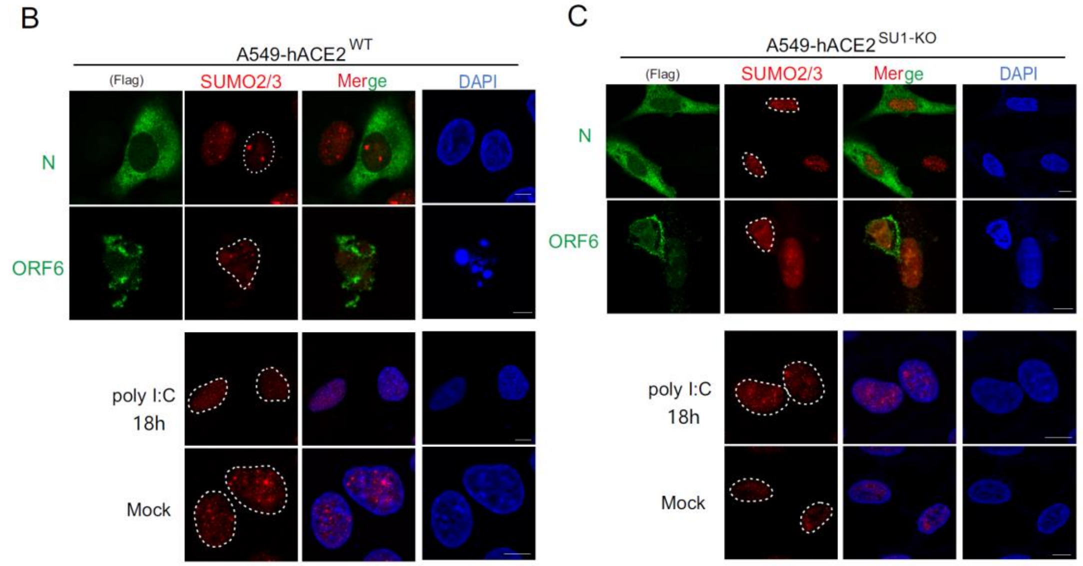


**Figure S2**. (**A**) The top three ranked viral proteins encoded by SARS-CoV-2 related to SUMO modification predicted by GPS-SUMO software. The entire genome sequence of SARS-CoV-2 WHU1 strain was subjected to GPS-SUMO (version 1.0) prediction tool (http://sumo.biocuckoo.cn/advanced.php) for SUMOylated sites and SIM motifs. (**B**) Overexpression of CoV-2-encoding N and ORF6 protein or Poly I: C treatment does not enhance SUMO2/3 perinuclear localization as puncta. A549-hACE2 WT or A549-hACE2 SU1-KO cells were individually transfected with the CoV-2-encoding N and ORF6 protein, or treated with Poly I: C for 18 h at 24h post-transfection, followed by immunofluorescence using antibodies against Flag tag and SUMO2/3. Mock is untreated group.

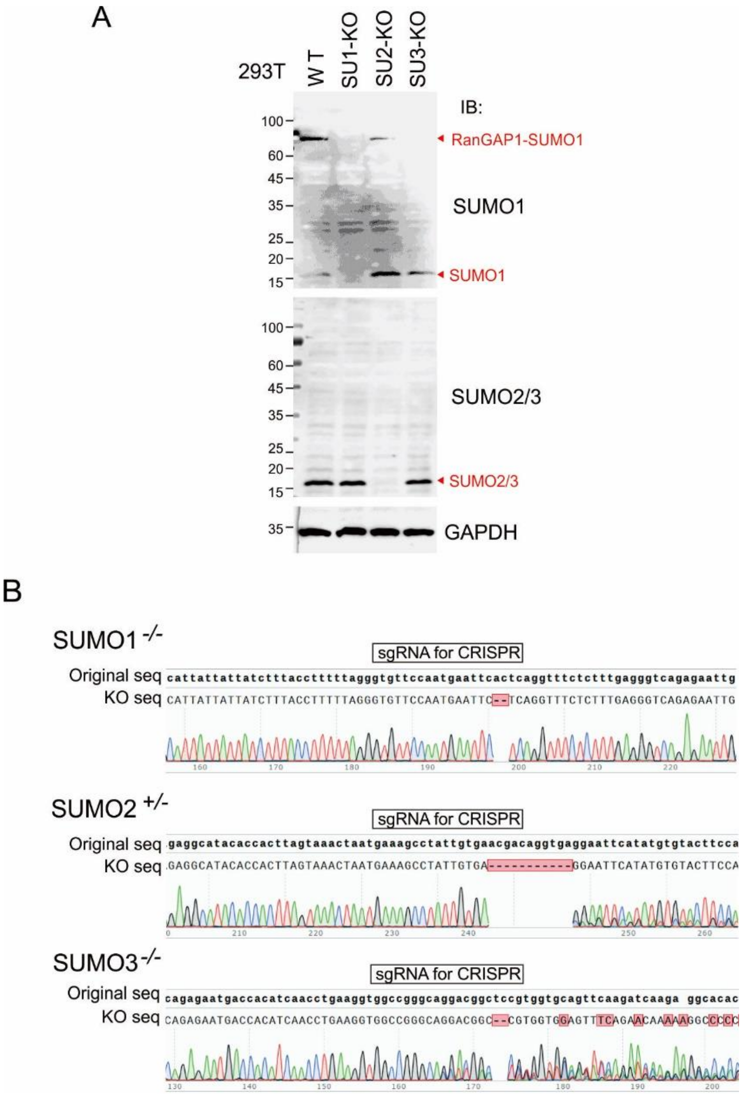


**Figure S3**. (**A**) Expression level of SUMO protein in HEK293T SUMO-KO cell lines. Whole cell lysates from wild-type (WT) or SUMO-knockout (SU1-KO, SU2-KO, and SU3-KO) HEK293T cells were subjected to immunoblotting using the antibodies indicated in the figure. (B) Validation of SUMO1/2/3 gene knockout in the HEK293T SUMO-KO cell lines. Total DNA was extracted from HEK293T SU1-KO, SU2-KO and SU3-KO cell lines, reverse-transcribed, and second-generation sequencing. The image shows the alignment of small guide RNA (sgRNA) targeting sequences

for CRISPR-Cas9 with the original genomic sequences, confirming the successful knockout of the SUMO1/2/3 genes.

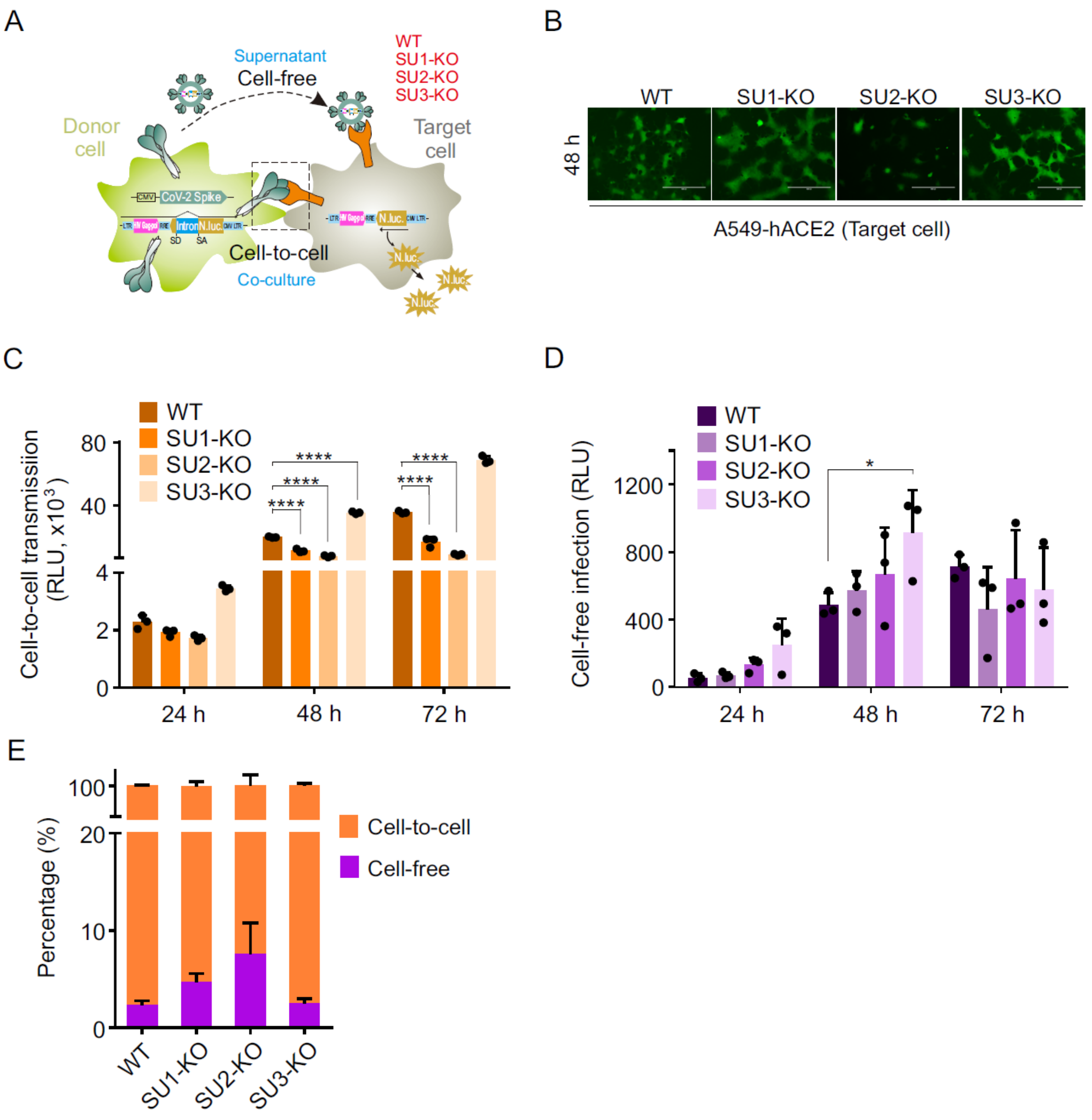


**Figure S4**. SUMO2 KO in target cells reduces viral cell-to-cell transmission. (**A**) Schematic representation of SUMO modification's effect of Spike protein on cell-to-cell transmission. Donor HEK293T cells were co-transfected with plasmids encoding the Spike protein, an NL4.3-inNluc reporter, and eGFP. At 24 h post-transfection, donor cells were co-cultured with target cells A549-hACE2 either wild-

type (WT) or SUMO KO (SU1-KO, SU2-KO, SU3-KO) for 72 h, after which luciferase activity was measured. For cell-free infection, virus was harvested from an equivalent number of transfected donor cells and used to infect equal numbers of A549-hACE2 cells and untransfected donor cells. (**B**) SUMO2 KO in target cells reduces Spike-mediated syncytia formation. A representative image of immunofluorescence analysis of donor cells at 48 h post-co-culture with target cells (as described in panel **A**). (**C**) SUMO2 KO in target cells dominantly reduces Spike-mediated cell-to-cell transmission. Data is from three independent experiments in panel **A**. (**D**) SUMO3 KO in target cells exclusively induces Spike-mediated cell-free infection. Three independent experiments in panel **A** show that SUMO3 KO specifically enhances cell-free infection mediated by the Spike protein. (**E**) SUMO2 KO in target cells dominantly reduces the ratio of cell-to-cell to cell-free infection mediated by the Spike protein. The relative ratio of cell-to-cell to cell-free infection was calculated from data in panels **C** and **D**. Each bar represents the mean ± standard deviation. **$p$< 0.01; *** $p$< 0.001; and **** $p$ < 0.0001.

**A**

| Position | Peptide | Score | Cut-off | Type |
|---|---|---|---|---|
| 510-514 | VGYQPYR**VVVLS**FELLHAP | 0.9973 | 0.85 | SUMO interaction |
| 1176-1180 | ISGINAS**VVNIQ**KEIDRLN | 0.9653 | 0.85 | SUMO interaction |
| 117-121 | LDSKTQS**LLIVN**NATNVVI | 0.8973 | 0.85 | SUMO interaction |
| 126-130 | IVNNATN**VVIKV**CEFQFC | 0.8602 | 0.85 | SUMO interaction |
| 1264-1268 | DEDDSEP**VLKGV**KLHYT** | 0.8586 | 0.85 | SUMO interaction |
| 1269 | EPVLKGV**K**LHYT*** | 0.7903 | 0.72 | SUMOylation |
| 1149 | QPELDSF**K**EELDKYF | 0.7737 | 0.72 | SUMOylation |
| 986 | DILSRLD**K**VEAEVQI | 0.7502 | 0.72 | SUMOylation |
| 129 | NATNVVI**K**VCEFQFC | 0.746 | 0.72 | SUMOylation |
| 462 | LFRKSNL**K**PFERDIS | 0.7293 | 0.72 | SUMOylation |
| 933 | QFNSAIGKIQDSLSS | 0.7208 | 0.72 | SUMOylation |

**B**

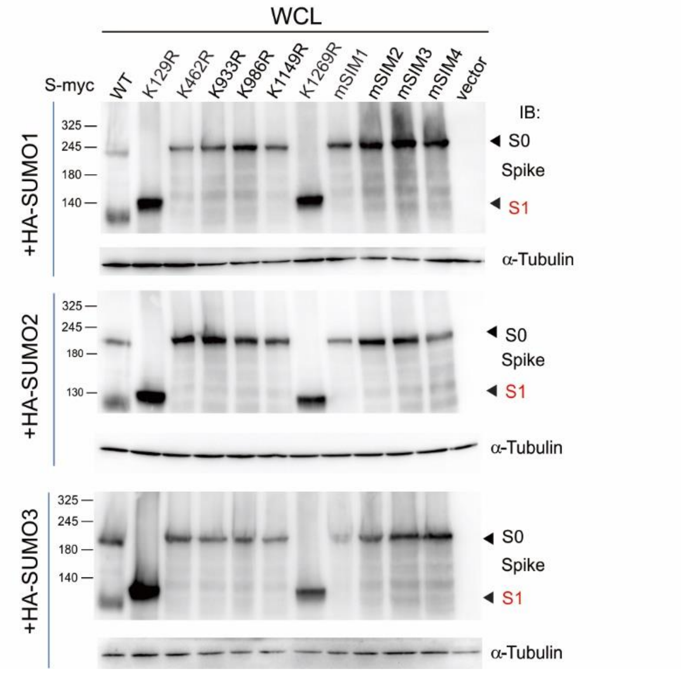


**Figure S5**. (**A**) The potential SUMOylated sites and SIM motifs of Spike proteins predicted by GPS-SUMO. The amino acid sequence of Spike protein encoded by SARS-CoV-2 WHU1 strain was subjected to analyze for SUMOylated sites and SIM motifs by using the GPS-SUMO (version 2.0) tool (http://sumo.biocuckoo.cn/advanced.php). (**B**) HEK293T cells were transfected

with myc-tagged WT Spike or its mutants, either in the presence or absence of HA-tagged SUMO. At 36 h post-transfection, cells were treated with MG132 for 12 h before being harvested for directly immunoblotting assays using the antibodies against Spike (600-700). $\alpha$-tubulin was used as an internal control.

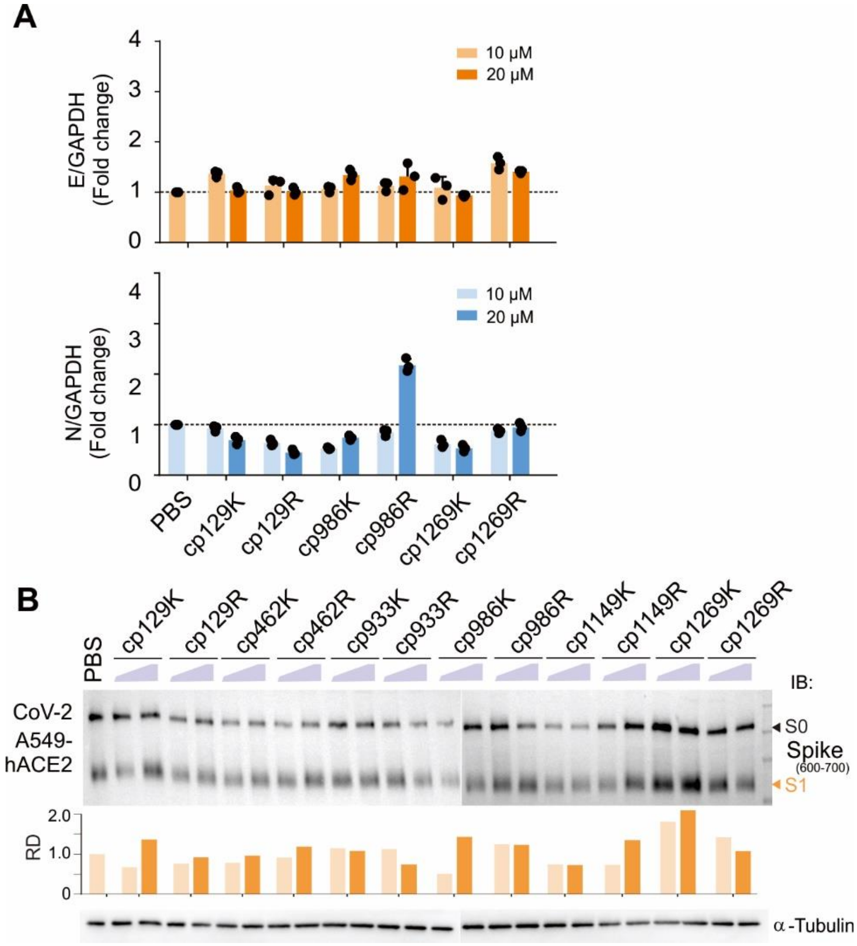


**Figure S6**. The cp129K/R peptides derived from the SUMOylated sites K129 of Spike protein dramatically promotes SARS-CoV-2 replication. (**A**) The relative viral replication within host cells were analyzed by qPCR targeting the CoV-2 N or E gene. All analyses were conducted using the same A549-hACE2 cells as in Figure 6A. (**B**) Immunoblotting was performed on cell lysates from panel B using antibodies against the Spike protein (600-700). The relative density (RD) of the cleaved Spike form (S1) band was quantified and is shown in the middle panel. The $\alpha$-tubulin served as an internal control.

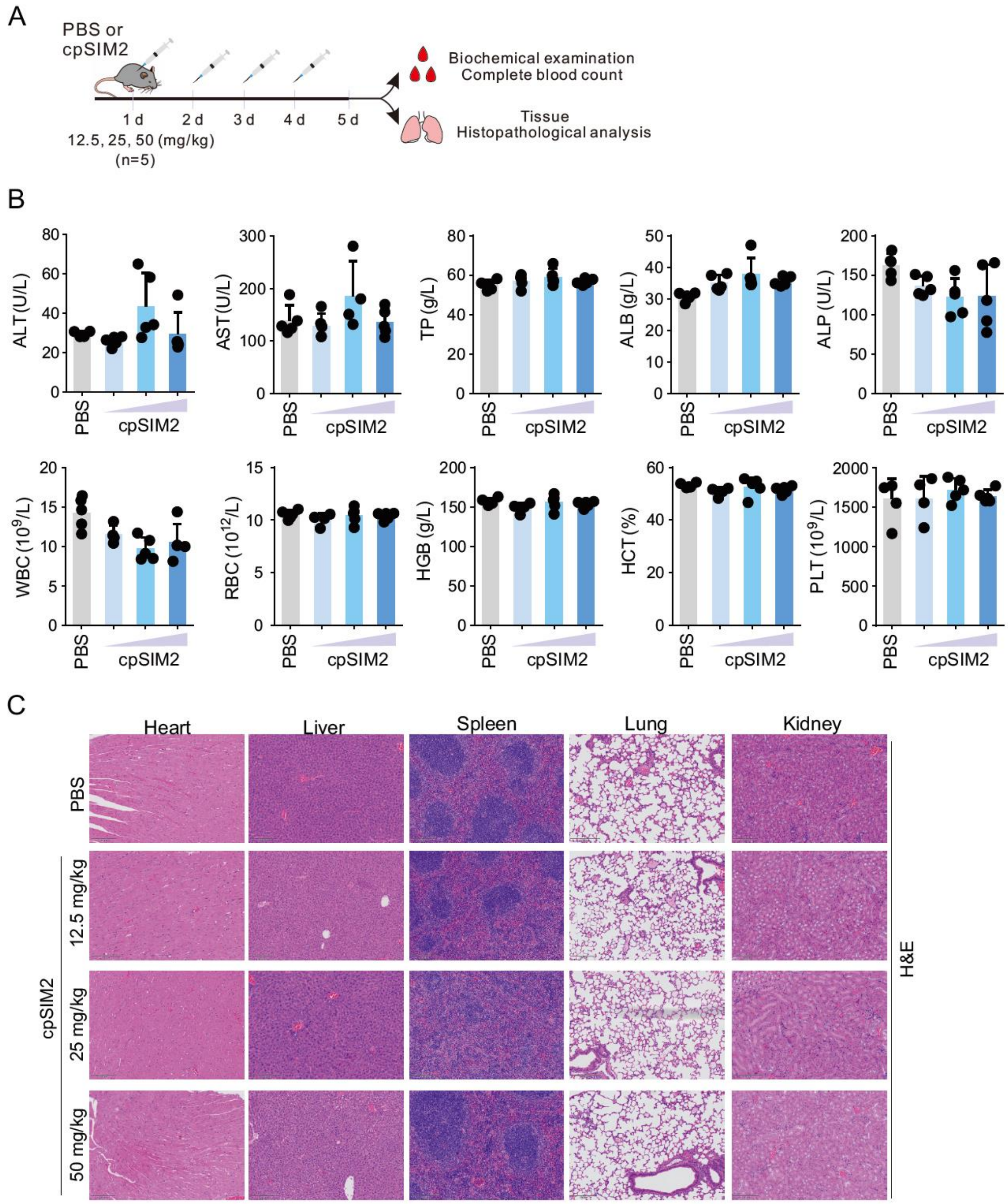


**Figure S7**. Toxicity profiles of cpSIM2 peptide in mice.

(**A**) Schematic of cpSIM2 peptide injection in mice. C57BL/6 mice (n=5) were intraperitoneally injected with PBS or cpSIM2 at dosage of 12.5, 25, or 50 mg/kg daily for 4 days. On day 5 post-treatment, mice were euthanized, and blood and

organ tissues were individually collected for analysis. (**B**) Comparison of blood parameters. Blood samples were analyzed for biochemical and hematological parameters, including alanine aminotransferase (ALT), aspartate aminotransferase (AST), total protein (TP), albumin (ALB), alkaline phosphatase (ALP), white blood cell (WBC), red blood cell (RBC), hemoglobin (HGB), hematocrit (HCT), and platelet (PLT) counts. Data are presented as mean ± SD (n=5). (**C**) Histopathological analysis. Representative H&E-stained sections of major organs (liver, kidney, heart, spleen, and lung) were prepared to evaluate tissue morphology and potential pathology. Staining was performed twice independently with consistent results.

**Table S1**. Oligo sequence for plasmid construction

| Primer | Oligo Sequence |
|---|---|
| S-pA3M-F | 5'-CAAGCTTGGTACCGAGCTCGGCCACCATGTTCGTGTTCCTGGT-3' |
| S-pA3M-R | 5'-GCCAGTGTGATGGATATCTGCAGGGTGTAGTGCAGCTTCACGC-3' |
| K129R-F | 5'-CAACGCTACAAATGTGGTGATCAGGGTGTGCGAGTT-3' |
| K129R-R | 5'-AACTCGCACACCCTGATCACCACATTTGTAGCGTTG-3' |
| K462R-F | 5'-ACTGTTCAGAAAGAGCAATCTGAGGCCTTTCGAGAG-3' |
| K462R-R | 5'-CTCTCGAAAGGCCTCAGATTGCTCTTTCTGAACAGT-3' |
| K933R-F | 5'-AGCGCCATCGGCAGGATCCAGGACAGC-3' |
| K933R-R | 5'-GCTGTCCTGGATCCTGCCGATGGCGCT-3' |
| K986R-F | 5'-CTGAGCAGACTGGACAGGGTGGAGGCCG-3' |
| K986R-R | 5'-CGGCCTCCACCCTGTCCAGTCTGCTCAG-3' |
| K1149R-F | 5'-CAGCTCCTCCCTGAAGCTGTCCAGCTCAGG-3' |
| K1149R-R | 5'-CCTGAGCTGGACAGCTTCAGGGAGGAGCTG-3' |
| K1269R-F | 5'-CTGTGCTGAAGGGCGTGAGGCTGCACTAC-3' |
| K1264R-R | 5'-TGTGATGGATATCTGCAGGGTGTAGTGCAGCCTC ACGCCCTT-3' |
| mSIM1-F | 5'-CACCACCTTGGATTCCAAGACTCAGAGCGCGGCGGC TGCAAACAACGCTACAAATGTGGTGATCAAG-3' |
| mSIM1-R | 5'-CTTGATCACCACATTTGTAGCGTTGTTTGCAGCCGCCGCG CTCTGAGTCTTGGAATCCAAGGTGGTG-3' |
| mSIM1'-F | 5'-GCTGATTGTAAACAACGCTACAAATGCGGCGGCCGCGG CGTGCGAGTTCCAGTTCTGCAATGACC-3' |
| mSIM1'-R | 5'-GGTCATTGCAGAACTGGAACTCGCACGCCGCGGCC GCCGCATTTGTAGCGTTGTTTACAATCAGC-3' |
| mSIM2-F | 5'-AGCGTGCAGCAGCTCGAAGGCCGCCGCCGCCGCTCTG TAAGGCTGGTAGCC-3' |
| mSIM2-R | 5'-GGCTACCAGCCTTACAGAGCGGCGGCGGCGGCCTTCG AGCTGCTGCACGCT-3' |
| mSIM3-F | 5'-TTCGGGAATCAATGCCAGCGCGGCGAATGCCCAGAAGGA |

| | |
|---|---|
| | AATTGATCGGC-3' |
| mSIM3-R | 5'-GCCGATCAATTTCCTTCTGGGCATTCGCCGCGCTGGCATTG ATTCCCGAA-3' |
| mSIM4-F | 5'-GACAGCGAGCCTGCGGCGAAGGGCGCGAAGCTGCACTAC-3' |
| mSIM4-R | 5'-GTAGTGCAGCTTCGCGCCCTTCGCCGCAGGCTCGCTGTC-3' |
| ORF9-pA3F-F | 5'-CTGGCTAGCGTTTAAACTTAATGGACCCCAAAATCAGCGA-3' |
| ORF9-pA3F-R | 5'-AGTGTGATGGATATCTGCAGTTTTACCGTCACCACCACGA-3' |

**Table S2**. Oligo sequence for qPCR

| Primers | Oligo sequence |
|---|---|
| N qPCR Forward | 5′-GACCCCAAAATCAGCGAAAT-3′ |
| N qPCR Reverse | 5′-CTGGTTACTGCCAGTTGAATCTG-3′ |
| E qPCR Forward | 5′-CGATCTCTTGTAGATCTGTTCTC-3′ |
| E qPCR Reverse | 5′-ATATTGCAGCAGTACGCACACA-3′ |
| GAPDH qPCR Forward | 5′-TGCCTTCTTGCCTCTTGTCT-3′ |
| GAPDH qPCR Forward | 5′-GGCTCACCATGTAGCACTCA-3′ |

**Table S3**. Biochemical and hematological parameters of cpSIM2 peptide in mice

| Group | Number | TBIL (μM) | TBA (μM) | Urea (mM) | Crea (μM) | Ca2+ (mM) | P (mM) | TC (mM) | TG (mM) | CK (U/L) | GLOB (g/L) |
|---|---|---|---|---|---|---|---|---|---|---|---|
| PBS | 1 | 4.44 | 146.1 | 6.50 | 31.6 | 2.61 | 2.55 | 1.26 | .95 | 1436.3 | 21.5 |
| | 2 | 2.28 | 6.2 | 8.99 | 33.5 | 2.70 | 2.67 | 2.12 | 1.18 | 1609.8 | 26.4 |
| | 3 | 1.10 | 3.9 | 9.92 | 27.0 | 2.71 | 2.74 | 2.25 | 1.38 | 2462.2 | 24.3 |
| | 4 | 1.58 | 3.4 | 7.34 | 28.7 | 2.56 | 3.00 | 2.15 | 1.15 | 1587.4 | 24.5 |
| | 5 | 1.77 | 6.2 | 8.39 | 40.7 | 2.62 | 2.40 | 2.26 | 1.42 | 2248.6 | 24.6 |
| CpSIM2 12.5 mg/kg | 1 | 0.6 | 8.4 | 6.9 | 28.6 | 2.6 | 3.0 | 2.8 | 1.9 | 2750.8 | 21.4 |
| | 2 | -0.2 | 9.0 | 6.3 | 29.0 | 2.6 | 2.9 | 2.6 | 1.6 | 1774.6 | 21.4 |
| | 3 | 1.3 | 10.8 | 7.0 | 29.2 | 2.6 | 2.8 | 2.4 | 1.2 | 1673.2 | 20.8 |
| | 4 | 1.3 | 6.2 | 7.0 | 30.0 | 2.5 | 2.4 | 2.2 | 1.1 | 1418.8 | 20.8 |
| | 5 | 0.2 | 9.5 | 7.5 | 31.0 | 2.6 | 2.5 | 2.6 | 1.6 | 2720.8 | 21.3 |

| | | | | | | | | | | | |
|---|---|---|---|---|---|---|---|---|---|---|---|
| CpSIM2 25 mg/kg | 1 | -0.9 | 11.5 | 5.2 | 44.5 | 2.7 | 3.9 | 2.4 | 1.3 | 4814.0 | 19.0 |
| | 2 | 0.4 | 5.0 | 8.4 | 37.4 | 2.6 | 3.6 | 2.2 | 1.2 | 5547.0 | 20.2 |
| | 3 | 1.6 | 8.2 | 5.6 | 29.6 | 2.6 | 2.5 | 2.1 | 1.0 | 2358.0 | 22.2 |
| | 4 | 0.9 | 5.6 | 6.4 | 40.2 | 2.6 | 2.8 | 2.2 | 1.0 | 2246.0 | 23.2 |
| | 5 | 1.0 | 5.6 | 5.8 | 27.6 | 2.6 | 2.6 | 2.3 | 0.9 | 1644.0 | 21.6 |
| CpSIM2 50 mg/kg | 1 | 3.6 | 191.6 | 4.2 | 26.0 | 2.6 | 4.1 | 1.6 | 1.2 | 2375.2 | 18.2 |
| | 2 | 2.21 | 8.1 | 8.01 | 36.7 | 2.75 | 2.17 | 2.27 | 1.02 | 1664.9 | 27.0 |
| | 3 | 2.5 | 14.4 | 7.0 | 27.6 | 2.7 | 2.7 | 2.5 | 1.2 | 1821.0 | 18.8 |
| | 4 | 2.6 | 6.6 | 5.6 | 31.8 | 2.7 | 2.5 | 2.2 | 1.0 | 2090.6 | 20.8 |
| | 5 | 1.92 | 8.0 | 8.29 | 32.9 | 2.77 | 2.49 | 2.20 | 1.00 | 1238.8 | 25.7 |
